# Genetic Analysis of Fin Development in Zebrafish Identifies Furin and Hemicentin1 as Potential Novel Fraser Syndrome Disease Genes

**DOI:** 10.1371/journal.pgen.1000907

**Published:** 2010-04-15

**Authors:** Thomas J. Carney, Natália Martins Feitosa, Carmen Sonntag, Krasimir Slanchev, Johannes Kluger, Daiji Kiyozumi, Jan M. Gebauer, Jared Coffin Talbot, Charles B. Kimmel, Kiyotoshi Sekiguchi, Raimund Wagener, Heinz Schwarz, Phillip W. Ingham, Matthias Hammerschmidt

**Affiliations:** 1 Max-Planck Institute of Immunobiology, Georges-Koehler-Laboratory, Freiburg, Germany; 2 Institute of Molecular and Cell Biology, Proteos, Singapore; 3 Institute of Developmental Biology, University of Cologne, Cologne, Germany; 4 Center for Molecular Medicine Cologne, University of Cologne, Cologne, Germany; 5 Institute for Protein Research, Osaka University, Osaka, Japan; 6 Center for Biochemistry, University of Cologne, Cologne, Germany; 7 Institute of Neuroscience, University of Oregon, Eugene, Oregon, United States of America; 8 Max-Planck Institute of Developmental Biology, Tübingen, Germany; 9 Cologne Excellence Cluster on Cellular Stress Responses in Aging-Associated Diseases, University of Cologne, Cologne, Germany; University of Pennsylvania School of Medicine, United States of America

## Abstract

Using forward genetics, we have identified the genes mutated in two classes of zebrafish fin mutants. The mutants of the first class are characterized by defects in embryonic fin morphogenesis, which are due to mutations in a Laminin subunit or an Integrin alpha receptor, respectively. The mutants of the second class display characteristic blistering underneath the basement membrane of the fin epidermis. Three of them are due to mutations in zebrafish orthologues of FRAS1, FREM1, or FREM2, large basement membrane protein encoding genes that are mutated in mouse bleb mutants and in human patients suffering from Fraser Syndrome, a rare congenital condition characterized by syndactyly and cryptophthalmos. Fin blistering in a fourth group of zebrafish mutants is caused by mutations in Hemicentin1 (Hmcn1), another large extracellular matrix protein the function of which in vertebrates was hitherto unknown. Our mutant and dose-dependent interaction data suggest a potential involvement of Hmcn1 in Fraser complex-dependent basement membrane anchorage. Furthermore, we present biochemical and genetic data suggesting a role for the proprotein convertase FurinA in zebrafish fin development and cell surface shedding of Fras1 and Frem2, thereby allowing proper localization of the proteins within the basement membrane of forming fins. Finally, we identify the extracellular matrix protein Fibrillin2 as an indispensable interaction partner of Hmcn1. Thus we have defined a series of zebrafish mutants modelling Fraser Syndrome and have identified several implicated novel genes that might help to further elucidate the mechanisms of basement membrane anchorage and of the disease's aetiology. In addition, the novel genes might prove helpful to unravel the molecular nature of thus far unresolved cases of the human disease.

## Author Summary

There are a large number of human genetic syndromes with limb and digit deformities. It has been shown that the genes underlying these syndromes are well conserved in evolution, and most perform the same role even in the fins of fish. One such human syndrome is Fraser Syndrome, characterized by a number of defects including fusion of the fingers (syndactyly). Data obtained with corresponding mouse mutants suggest that all of these defects are due to transient basement membrane disruptions and epithelial blistering during development. Whilst some of the Fraser Syndrome genes have been identified, others are unknown. We show that mutation of the known Fraser Syndrome genes in zebrafish generate comparable blistering defects in the fins. Importantly, we have also identified additional genes and mechanisms required for the same processes. Included in this are *hemicentin1*, a gene whose function had thus far only been studied in nematodes, and furinA, encoding a proprotein convertase, for which we reveal a novel role in ectodomain shedding of Fras/Frem proteins. This work thus expands our understanding, not only of Fraser Syndrome, but also of the common processes of basement membrane formation and function during fin and limb development.

## Introduction

Fraser Syndrome (FS) is a recessive polygenic, multisystem congenital human disorder characterised largely by syndactyly of the soft tissue of the digits, cryptophthalmos (fusion of the eye lids) and renal agenesis, although a myriad of other variable epithelial malformations have been reported, underscoring the complex and pleiotropic nature of the syndrome [1]. Autozygosity mapping and candidate sequencing revealed that many Fraser syndrome cases are due to mutations in the genes encoding the proteins FRAS1 or FREM2, which belong to a family of large extracellular matrix proteins [2], [3], [4]. This protein family contains two further members, FREM1 and FREM3, however these have not, so far, been implicated in Fraser Syndrome aetiology [5], [6]. Our understanding of the molecular function of the FRAS1 and FREM protein family has been aided by analysis of four mouse ‘bleb’ mutants [reviewed in 7]. The phenotypes of these mutants are strikingly similar to the malformations seen in Fraser patients and have long been considered to represent murine equivalents of Fraser syndrome [8]. Indeed the ‘bleb’ mouse mutants have recently been shown to correspond to mutations in the genes encoding Fras1 [3], [4], Frem2 [2] and Frem1 [6], as well as the intracellular trafficking protein Grip1, required for correct basal localisation of the Fras1 and Frem2 proteins [9]. The embryonic expression domains of the Fras/Frem complex during development coincide with sites later disrupted in the bleb mutants, including the eye, the apical ectodermal ridges of the limb buds and the kidney. Immunogold-labelling localised the proteins to the basement membranes, consistent with the embryonic epidermal blistering and other defects [2], [4], [10], [11]. As the blisters occur below the lamina densa, it has been suggested that the Fras/Frem proteins mediate adhesion of the basement membrane to the underlying dermis [reviewed in 12]. Aside from the interactions demonstrated between the Fras/Frem family members, other ECM components to which the complex binds are unknown. Identification of these interactions will elucidate the precise role the Fras/Frem complex plays in maintaining adhesion. Furthermore, approximately 50% of the Fraser Syndrome patients have no mutation in any of the candidate genes described, indicating that other unidentified loci contribute to Fraser Syndrome.

Here, based on the genetic analysis of fin development in the zebrafish [13], we identify several additional potential Fraser syndrome disease genes. Teleosts possess two types of fins; the paired fins including the pelvic and pectoral fins (homologues of tetrapod hindlimbs and forelimbs respectively), and unpaired or medial fins consisting of the dorsal, tail (caudal) and anal fins. Whilst paired fins and appendages form from buds found at two axial positions on the ventrolateral trunk, the medial fins are derived from an initial continuous fin fold generated along the midline at embryonic stages [14], [15]. This fin fold is comprised of two apposed sheets of bilayered epidermis, between which are found numerous extracellular matrix structures including two basement membranes, rod-like collagenous fibers called actinotrichia, and extracellular cross fibres [16]. Outgrowth of both fin types is mediated by the induction of a signalling structure, the apical ectodermal ridge that is also present during tetrapod limb growth [16], [17].

To identify the molecules required for adhesion of the epidermis during zebrafish fin outgrowth, we applied a combination of chromosomal mapping, positional cloning and candidate testing of ENU-induced mutations [13], revealing six essential proteins: Lamininα5, Integrinα3, zebrafish orthologues of Fras1, Frem1, Frem2, and Hemicentin1 (Hmcn1), the latter being an ECM protein with hitherto unknown function in vertebrate biology. Morphologically, and with respect to synergistic interactions, the mutants fall into two classes, with Fras1, Frem1, Frem2 and Hmcn1 displaying a characteristic formation of fin blisters at the level of the lamina densa of the basement membrane, reminiscent of the blistering seen in the limb buds of the mouse bleb mutants. Very similar phenotypes and dose-dependent interactions were obtained upon antisense-mediated loss of zebrafish orthologues of the other mouse bleb genes (Grip1/2) and the ECM protein Fibrillin2 [18], and upon mutations in the proprotein convertase FurinA (*sturgeon*) [19]. Biochemical analyses further implicate Furin in the proteolytic shedding of Fras1 and Frem2 from the cell membrane. Together, we demonstrate that the zebrafish is a useful model for elucidating mechanisms and novel players involved in Fraser Syndrome, and that the Fraser complex is an ancient invention with essential roles during the formation and/or function of basement membranes in particular epithelial structures of the developing embryo.

## Results

### Zebrafish fin mutants fall into two main phenotypic classes

**Figure 1:**
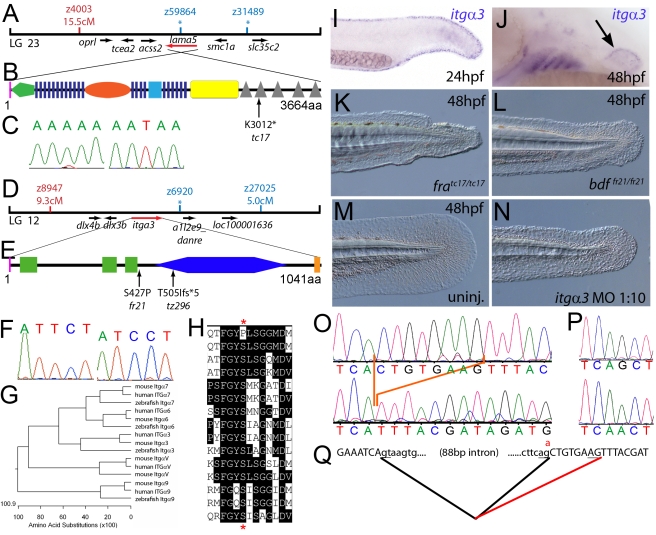
The fin dysmorphogenesis of *fransen* and *badfin* mutants are caused by mutations in Lamininα5 and Integrinα3 subunits. (A) Linkage analysis localised the *fransen* locus to LG23 close to the SSLP marker z59864, a region containing the *lama5* gene. Here and in subsequent figures, all markers north of the locus are represented in red, whilst those south in blue. Approximate genetic distances of markers relative to the mutated locus (in centi Morgan), calculated by Kosambi's mapping function, are given below each marker, with non-recombining markers indicated by an asterisk. Location and relative orientation of candidate genes residing in the interval are depicted below the map as arrows, with the affected gene coloured red. (B) Schematic of Lamininα5 protein showing the domains of the protein, namely the signal peptide (pink bar), a laminin N-terminal domain (green pentagon), laminin-type EGF-like domains (dark blue bars), an LF-like domain (orange oval), a laminin B domain (light blue box), a coiled-coil domain (yellow box) and 5 laminin G domains (grey triangles). The location and molecular nature of the *fransen^tc17^* mutation is given below. (C) Sequence chromatograms of *lama5* cDNA from *fra^tc17/tc17^* mutant (right panel) and WT sibling (left panel). (D) Linkage analysis localised the *bdf^fr21/fr21^* mutant to LG 12, near z6920. Candidate genes in the region were tested and *itga3* was further characterised. (E) Protein schematic of Itgα3 protein showing the signal peptide (pink bar), 3 Integrin β-propellers (green boxes), an Integrin alpha domain (blue hexagon) and a transmembrane domain (orange bar). The location and details of the *bdf^fr21^* and *bdf^tz296^* molecular lesions are given. (F) Sequence chromatograms of *itga3* cDNA from *bdf^fr21/fr21^* mutant (right panel) and WT sibling (left panel). (G) Phylogenetic tree of 5 different Integrin proteins from human, mouse and zebrafish showing that the integrin mutated in *bdf* is a true Itgα3 orthologue. (H) Protein ClustalW alignment showing that the *bdf^fr21^* mutation occurs in a residue (red asterisks) conserved across multiple Integrins in disparate vertebrates. Sequences are from top, zebrafish Itgα3 from *bdf^fr21/fr21^* mutant, wild-type zebrafish Itgα3, human Itgα3, mouse Itgα3, human ItgαV, mouse ItgαV, zebrafish ItgαV, human Itgα6, mouse Itgα6, zebrafish Itgα6, human Itgα7, mouse Itgα7, human Itgα9, mouse Itgα9, zebrafish Itgα7. (I–J) Lateral views of embryos stained by in situ hybridisation with a probe for *itga3* showing expression in the medial fin fold at 24 hpf (I) and in the pectoral fin (arrow) at 48 hpf along with expression in the branchial arches and neuromasts (J). (K–N) Lateral Normaski images of the medial fin of a *fra^tc17/tc17^* embryo (K), a *bdf^fr21/fr21^* embryo (L), or an embryo injected with a MO targeting the translation start of the *itga3* mRNA at 48 hpf (N). The *itga3* morphant displays moderate dysmorphogenesis of the fin, highly reminiscent of the *bdf^fr21/fr21^* mutant at this stage and in contrast to uninjected control (M). (O–Q) Sequence chromatograms of *itga3* cDNA of *bdf^tz296/tz296^* mutant (O; lower panel), and WT sibling (O; upper panel), showing the deletion of 8 nucleotides in the mutant cDNA, and of *itga3* genomic DNA of *bdf^tz296/tz296^* mutant (P; lower panel) and WT sibling (P; upper panel), showing the mutation of the splice acceptor site. (Q) illustrates altered splicing at the exon 10 — exon 11 junction in *bdf^tz296/tz296^* mutant compared to WT sibling. Here and in subsequent figures, normal splicing is shown as black lines joining the exon sequences (represented in uppercase). The intron sequences are in lowercase with the normal or cryptic splice donor and acceptor sites underlined. The substituted base is shown in red above the WT base, with the mutated splice acceptor generating aberrant splicing with use of a cryptic splice acceptor (illustrated as a red line).

To elucidate the mechanisms required for generating fins, we analysed zebrafish fin mutants isolated in previous [13] or more recent ENU mutagenesis screens conducted in the Hammerschmidt laboratory. Two main phenotypic classes could be distinguished by morphological criteria. One class, consisting of two loci, *fransen* (*fra*) and *badfin* (*bdf*), was characterised by medial fins that appeared ragged from about 30 hours post fertilisation (hpf) and that became progressively dysmorphic, such that by 48 hpf the fin fold was much reduced compared to wild-type (WT) embryos (Figure 1K, 1L, 1M and Figure S1G, S1H, S1O, S1P, S1W, and S1X). The pectoral fins were also dysmorphic in both mutants and the yolk sac extension appeared thinner in *fra* at 48 hpf (Figure S2A, S2H, and S2I; data not shown). The *bdf* mutant phenotype appeared to be less severe than that of *fra*, and is homozygous viable, with a proportion of *bdf* homozygous adults displaying hypoplastic fins (compare Figure S2J with Figure S2N). *fra* homozygous larvae however die at approximately 11 days post fertilisation (dpf).

**Figure 2:**
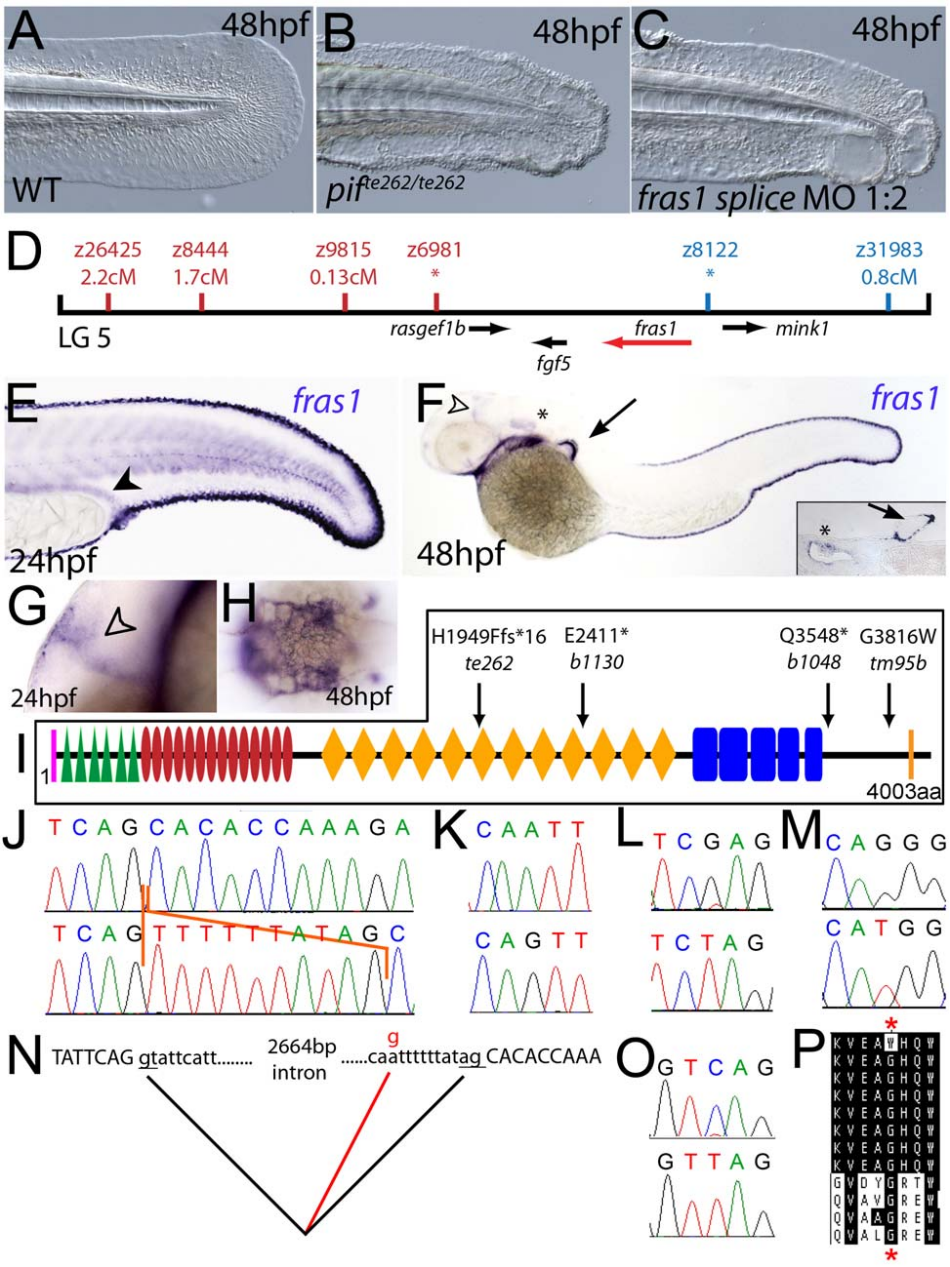
The fin blistering of *pinfin* mutants is caused by mutations in Fras1. (A–C) Injection of a morpholino targeting the translation site of *fras1* mRNA into wild-type embryos phenocopies the *pif* fin blisters; lateral views of tip of tail at 48 hpf; (A) uninjected control; (B) *pif^te262/te262^* mutant; (C) *fras1* morphant. (D) Linkage analysis localised the *pinfin* locus to linkage group 5, close to the SSLP markers z6981 and z8122, neither of which recombined with the locus, and between which the *fras1* gene (depicted in red) was located. (E–H) Images of embryos stained by in situ hybridisation using a probe against *fras1.* Close up view of the tail region of a 24 hpf embryo (E) and a 48 hpf embryo (F) are shown. The *fras1* gene is expressed in the medial fin fold, the pectoral fin fold (arrow in F; also see inset), the pronephric ducts (filled arrowhead in E) and the otic vesicle (asterisks in F and insert). *fras1* expression is also noted in the midbrain-hindbrain region at 24 hpf and 48 hpf (open arrowhead in G and F) and in the pharyngeal pouches of the arches (H). (E–G) show lateral views, (H) a dorsal view. (I) Schematic of the predicted domain structure of zebrafish Fras1 protein, with a signal peptide (pink bar), von Willebrand C domains (green triangle), furin-like domains (red ovals), CSPG domains (orange diamonds), Calx-bdomains (blue boxes) and a transmembrane domain (orange bar). Lesions found in four *pif* alleles are indicated above the protein schematic. (J,L,M,O) Sequence chromatograms of *fras1* cDNA from *pif^te262/te262^* (J), *pif^b1130/b1130^* (L), *pif^b1048/b1048^* (O), and *pif^tm95/tm95^* (M) mutants, displaying the lesions depicted in (I). The frame shift (fs) mutation *in pif^te262^* is due to the insertion of the last 10 nucleotides of intron 42 (delineated with orange lines). In all panels, the WT sibling chromatogram is given above the mutant chromatogram. (K) Chromatograms showing the genomic sequence at the end of intron 42 of the *fras1* gene in *pif^te262/te262^* (lower panel) and WT sibling (upper panel) embryos. The A>G substitution generates a novel splice acceptor. (N) Representation of the exon 42-exon 43 junction with the mutation generating aberrant splicing (red line). (P) The glycine residue (red asterisks) substituted in *pif^tm95/tm95^* is strictly conserved in both Fras1 and Frem2 proteins across diverse phyla, as seen in an alignment of the region. Protein sequences are from top: zebrafish *pif^tm95/tm95^* mutant Fras1; zebrafish wild-type Fras1; fugu Fras1; human FRAS1; mouse Fras1; dog Fras1; cow Fras1; chicken Fras1; sea urchin ECM3; zebrafish Frem2a; fugu Frem2a; human FREM2.

**Figure 3:**
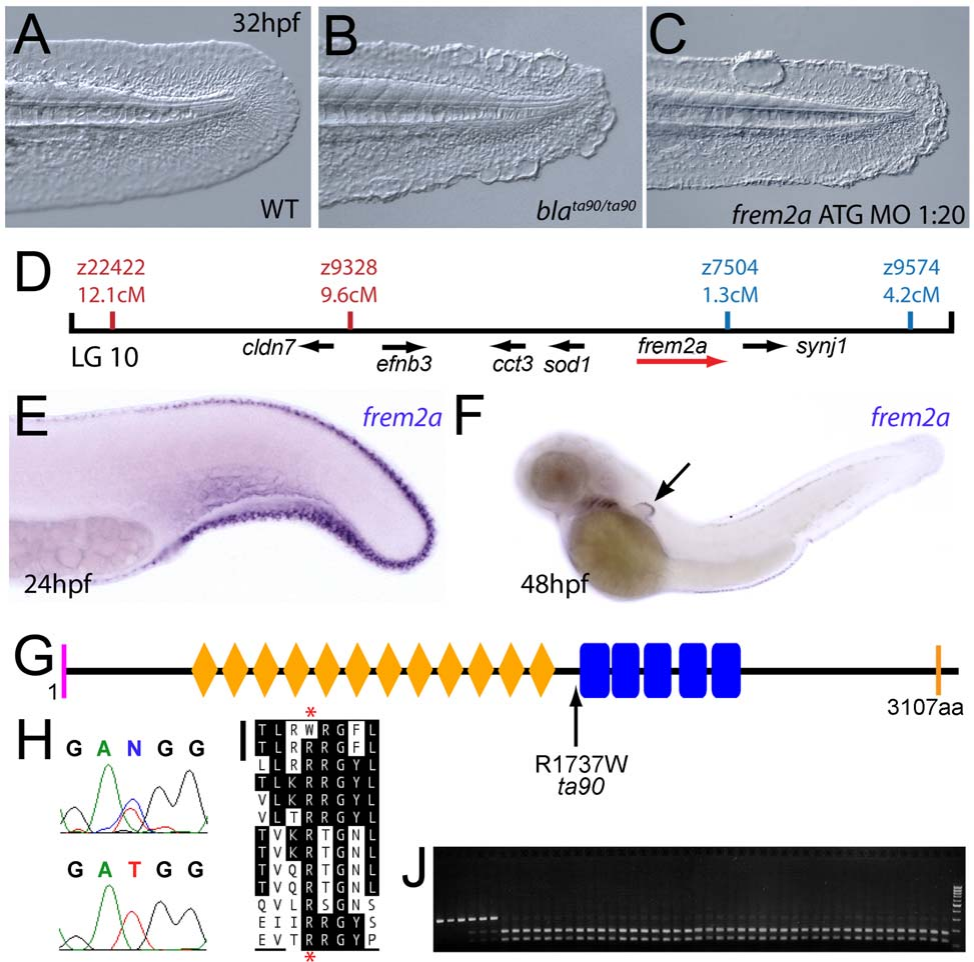
The fin blistering of the *blasen* mutant is caused by mutation of Frem2a. (A–C) Injection of a morpholino targeting the translation site of *frem2a* mRNA into WT embryos phenocopies the *bla* fin blisters; lateral views of posterior medial fin, 48 hpf; (A) uninjected control; (B) *bla^ta90/ta90^* mutant; (C) *frem2a* morphant. (D) Genetic map (set out as in Figure 1A), showing the approximate location of the *blasen* locus on linkage group 10 between markers z9328 and z7504, with genes within this interval shown below, including the strong candidate *frem2a* (red arrow). (E,F) Lateral views of embryos at 24 hpf (E) and 48 hpf (F) stained by in situ hybridisation for *frem2a,* revealing expression in the medial and pectoral fin fold (arrow, F). (G) Schematic of the zebrafish Frem2a protein with conserved domains as in Figure 2I, and the position and nature of the *bla^ta90^* mutation indicated. (H) Sequence chromatograms of *frem2a* cDNA from *bla^ta90/ta90^* mutants (lower panel) and heterozygous siblings (upper panel) showing the missense mutation depicted in (G). (I) Protein sequence alignment of Fras and Frem proteins showing the absolute conservation of the arginine residue mutated in *bla^ta90^* (red asterisks) across different vertebrate classes. Sequences from the top are: zebrafish *bla^ta90/ta90^* mutant Frem2a; zebrafish wild-type Frem2a; zebrafish Frem2b; zebrafish Frem3; human FREM2; mouse Frem2; zebrafish Fras1; fugu Fras1; human FRAS1; mouse Fras1; zebrafish Frem1a; human FREM1; mouse Frem1. (J) Segregation linkage analysis: the *bla^ta90^* mutation generates a *Bcc*I site, which is present in both alleles of all *bla* homozygotes (lanes 7–48), whilst wild-type siblings (lanes 1–6) show at least one copy of the uncut allele; lane 49, 100 bp ladder.

**Figure 4:**
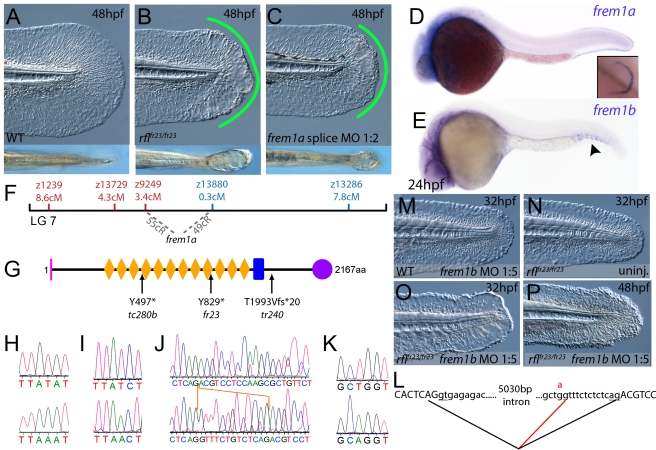
The fin blistering of *rafels* mutants is caused by mutations in Frem1a, which shows partial functional redundancy with Frem1b. (A–C) The *rafels* mutant displays mild blistering of the posterior fin at 48 hpf (B) compared to a sibling (A), and is phenocopied in wild-type embryos by injection of a morpholino targeting *frem1a* (C). Lower panels show dorsal views tail fin, providing a more striking image of the blistering in the mutant and morphant. (D,E) Expression of *frem1a* in the medial fin fold at 24 hpf (D) and pectoral fin at 48 hpf (D-inset). Expression of *frem1b* is weaker but can be seen in the tail region, in particular in the blood islands, at 24 hpf (E). (F) Genetic map (set out as in Figure 1A), showing the approximate location of the *rafels* locus on linkage group 7 between markers z9249 and z13880. Independent radiation hybrid mapping localised the *frem1a* gene to this region, with distances to the mapping markers given in grey. (G) Schematic of the zebrafish Frem1a protein with conserved domains as defined in Figure 2I. The purple circle depicts the C-type Lectin domain. The positions and natures of the molecular lesions of the *rfl^tc280b^*, *rfl^fr23^* and the *rfl^tr240^* alleles are indicated. (H–L) Sequence chromatograms of the mutations in the *frem1a* cDNA of *rfl^tc280b/tc280b^* (H), *rfl^fr23/fr23^* (I), and *rfl^tr240/tr240^* (J) (lower panels) compared to wild-type siblings (upper panels). The 13 nucleotide insertion in the *rfl^tr240/tr240^* allele is delineated by orange lines (J). Genomic sequencing of the intron32-exon33 boundary reveals generation of a novel splice site in the *rfl^tr240/tr240^* mutants (K; lower chromatogram), leading to aberrant splicing as depicted in (L) (red lines). (M–P) *frem1b* splice MO enhances the *rafels* phenotype. Lateral views of WT (M) or *rfl^fr23/fr23^* (N–P) embryos either uninjected (N) or injected with *frem1b* splice MO (M,O,P) photographed at 32 hpf (M–O) or 48 hpf (P). Whilst the *frem1b* MO does not generate a phenotype alone (M), it reveals a blistering phenotype in *rafels* mutants at 32 hpf (O), a time when a phenotype is not seen in uninjected mutants (N). The *frem1b* MO injected *frem1a* mutants often display degeneration of the fin at 48 hpf (P; compare to B).

The second class of mutants, consisting of *pinfin* (*pif*), *blasen* (*bla*), *rafels* (*rfl*) and *nagel* (*nel*), displayed characteristic temporary blistering within the medial fins, starting between 26 and 32 hpf (for *pif, bla* and *nel*) and noticeable at 48 hpf (Figure S1B, S1C, S1D, S1E, S1F, S1J, S1K, S1L, S1M, and S1N; Figure 2A and 2B; Figure 3A and 3B; Figure 4A and 4B; Figure 8A and 8B). However, blisters were no longer visible at 120 hpf, when the fin fold appeared slightly collapsed (Figure S1R, S1S, S1T, S1U, and S1V). These defects were also mirrored in the pectoral fins, albeit with a later onset, consistent with the later initiation of pectoral fin bud formation (Figure S2A, S2B, S2C, S2D, S2E, and S2G). There was a range of phenotypic severity among the different fin blister mutants and alleles (for details see legend of Figure S1). Blistering of the blood islands, leading to pooling of blood in the ventral fin was observed in all *pif* mutants and occasionally in *nel* mutants. *bla* was the least affected mutant, with blisters restricted to the tip of the tail fin, whilst *rfl* displayed moderately large blisters localised to the posterior portion of the medial fin. Uniquely *rfl* did not display blistering until 48 hpf, appearing indistinguishable from WT at 32 hpf (Figure S1E and S1M). With the exception of the 3 strongest *pif* alleles (*pif^b1130^*, *pif^b1048^*, and *pif^te262^*), which are lethal at around 10–12 dpf, all fin blister mutants were viable. The tail fin of adult homozyotes of the weak *pif* allele, *pif^tm95^*, was mis-patterned and had lost the bi-lobed structure (Figure S2J and S2K). In contrast, adult *bla, rfl* and *nel* mutants displayed no overt adult fin phenotype (Figure S2L, S2M, and S2N). The weak *pinfin* allele *pif^tm95^* was unique in that it showed a mild dominant larval phenotype, characterized by a single small blister in the medial fin fold (Figure S1Y and S1Z). Such combinations of partial loss-of-function (hypomorphic) with dominant negative effects, contrasting the purely recessive nature of amorphic (complete loss-of-function) alleles, have been previously also observed for other gene encoding proteins that act in homomeric complexes (see e.g. [20]).

### Compromised fin morphogenesis of *fransen* and *badfin* mutants is caused by mutations in Lamininα5 or Integrinα3, respectively

Meiotic mapping placed the *fra^tc17^* mutation in the vicinity of marker z59864 on linkage group 23 (Figure 1A), the same region to which the *m538* mutation in the *lamininα5* (*lama5*) gene has been recently mapped [21]. Sequencing the *lama5* coding region from cDNA made from *fra^tc17/tc17^* mutants (Genbank accession number GU936670) revealed an 9034A>T nonsense mutation, leading to a premature truncation of the protein at amino acid residue 3012 (Figure 1B and 1C) and a protein that lacks most of the C-terminal Laminin G domains required for receptor binding. Consistently, injection of a previously described antisense morpholino oligonucleotide (MO) directed against a splice site of the *lama5* gene (predicted to mimic the *fra^tc17^* mutation, also resulting in loss of the C-terminus of the protein [21]) yielded embryos displaying fin dysmorphogenesis as in *fra* mutants (data not shown). Together, this strongly suggests that *fra* represents an allele of *m538*, and that the fin dysmorphogenesis of *fra* mutants is caused by loss-of-function mutations in the *lamininα5* gene.

We next cloned the *bdf* mutation, which complements *fra* and thus represents another locus required for normal fin development. Rough mapping placed the mutation between markers z8947 and z27025 of LG 12, in the vicinity of z6920 (Figure 1D). The interval contains a gene encoding the zebrafish orthologue of Integrinα3 (*itga3*; Figure 1G; Genbank accession number GU936669), a subunit of the α3β1 dimer, a known receptor for the Lamininα5 containing Laminin511 heterotrimer [22]. Thus we considered *itga3* to be an excellent candidate for *bdf.* Indeed, in situ hybridisation revealed *itga3* expression in the median fin fold at 24 hpf, as well as in the pectoral fin at 48 hpf, sites affected in *bdf* mutants (Figure 1I and 1J). In addition, abolishing Itga3 levels through injection of wild-type embryos with MOs targeting either the translational start site of *itga3* mRNA or the splice donor site of exon 3, we obtained mild medial fin dysmorphogenesis (Figure 1N), reminiscent of the *bdf* phenotype (Figure 1L). Finally, we sequenced the *itga3* coding region from the two *bdf* alleles, *fr21* and *tz296*. The *bdf^fr21^* allele harboured a 1279T>C mutation in the coding region (Figure 1F), leading to a substitution of a serine residue that is conserved across many Integrin alpha subunits of multiple species (Figure 1E and 1H). The *bdf^tz296^* cDNA displayed a deletion of 8 nucleotides in the middle of the *itga3* coding region, resulting in a frameshift and predicted to result in the inclusion of 5 aberrant amino acids (IYDRC) and a premature termination of the protein directly before the integrin alpha domain (Figure 1E). Sequencing of genomic DNA further revealed that the deleted 8 nucleotides corresponded to the first 8 base pairs of exon 10, and that *bdf^tz296/tz296^* embryos had a G>A substitution at the final base of intron 10 (Figure 1P), abolishing the splice acceptor and forcing use of a cryptic splice acceptor within exon 11 (Figure 1Q). Taken together these data demonstrate that *itga3* is required for appropriate fin morphogenesis.

Due to the similarity of phenotype and their known direct physical interaction in vitro, we hypothesised that *itga3* and *lama5* might act synergistically in vivo. We tested this by co-injecting sub-phenotypic doses of MOs directed against both genes. Although individually, these MOs did not elicit a phenotype at these respective concentrations, co-injection generated embryos displaying compromised fin morphogenesis (Figure S3A, S3B, S3C, and S3D; Table 1) identical to that of *fra* (Figure 1K) or *bdf* (Figure 1L) mutants. This provides evidence that Itgα3 and Lamα5 function in the same pathway during zebrafish fin development in vivo, consistent with their physical interaction.

**Table 1:** Synergistic interaction between *itga3* and *lama5*.

	uninjected control		*itga3* ATG MO 1∶20		*lama5* splice MO 1∶150		*itga3* ATG MO 1∶20 + *lama5* splice MO 1∶150	
	C	S	C	S	C	S	C	S
WT fin (%)	100	100	96	96	100	100	61	36
single blister (%)	0	0	0	0	0	0	0	0
multiple blisters (%)	0	0	0	0	0	0	0	0
degenerate fins (%)	0	0	4	4	0	0	39	64
n	34	68	25	54	27	59	31	33

For each experimental treatment, numbers of embryos seen in the 4 phenotypic classes (WT fin, single blister, multiple blisters and degenerate fins) were assessed at 48 hpf. 1:xx values indicate injected dilution of 1 mM stock of used morpholino. Combined injections of morpholinos were performed in two ways, either co-injection of a mixture of morpholinos at the given doses, or sequential injection of the two morpholinos separately. Independent controls were done for both methods and each treatment is presented with two sub-columns of numbers for co-injection (C) and sequential injection (S). Numbers of embryos within one of the four different phenotypic categories (rows 1–4) are given in percent (%), total numbers of evaluated embryos per experiment (n) in row 5.

### The fin blistering of *pinfin*, *blasen*, and *rafels* mutants is caused by mutations in Fras1, Frem2a, and Frem1a, respectively

Chromosomal mapping approaches were also undertaken to determine the underlying genetic defects of the fin blister mutants. We mapped the *pif^tm95^* allele to LG5 between the markers z9815 and z31983 (Figure 2D). One of the genes within the corresponding interval was the zebrafish orthologue of the human Fraser syndrome gene FRAS1, mutations in which lead to similar epidermal blistering (see Introduction). Interestingly, the *bla^ta90^* mutation mapped to an interval of LG10 (between markers z9328 and z7504), which contains *frem2a*, a zebrafish homologue of *FREM2* (Figure 3D), the second Fraser syndrome gene in human. Concomitantly we localised the *frem1a* gene (an orthologue of FREM1) to LG7 via radiation hybrid mapping, noting that it co-mapped to the region corresponding to the *rafels* fin blistering mutant (Figure 4F). Whole mount in situ hybridisations revealed prominent expression of zebrafish *fras1, frem2a* and *frem1a* in the apical region of the median fin fold epithelium at 24 hpf, before the fin phenotype becomes apparent in *pif*, *bla* and *rfl* mutants (Figure 2E, Figure 3E, and Figure 4D). In addition, these genes were expressed in the apical ridge of the pectoral fin and in the pharyngeal arch region (Figure 2F and 2H; Figure 3F; Figure 4D). *fras1* additionally showed expression in the hypochord, somites, pronephric ducts and midbrain-hindbrain region at 24 hpf (Figure 2E and 2G), whilst also being expressed in the ear at 48 hpf (Figure 2F).

By sequencing *fras1* cDNA (Genbank accession number GU936658) from four different *pif* alleles, *frem2a* cDNA (Genbank accession number GU936661) from the single *bla* allele and *frem1a* cDNA (Genbank accession number GU936659) from three *rfl* alleles, we identified molecular lesions leading to premature truncations of the corresponding proteins, or the substitution of evolutionary conserved amino acid residues. *pif^b1130^* displayed a 7231G>T mutation in the *fras1* coding region, resulting in a premature translational termination after amino acid residue 2410, and *pif^b1048^* contained a 10642C>T transversion generating a premature stop codon at amino acid residue 3548 (Figure 2I, 2L, and 2O). *pif^te262^* mutants showed an A to G transversion in intron 42, 11 bp upstream of the normal start of exon 43, generating a new and preferentially used splice acceptor site. Accordingly, cDNA from mutant embryos contained an insertion of the last 10 base pairs of intron 42, leading to a frame shift and an inclusion of 16 aberrant amino acids (FFIAHQRGPSSNYLCK), followed by a stop codon, at amino acid residue 1949 (Figure 2I, 2J, and 2N). Finally, *pif^tm95b^* displayed a 11446G>T missense mutation, leading to the substitution of a totally conserved glycine residue at amino acid 3816 with a tryptophan (Figure 2I, 2M, and 2P). Similarly, sequencing the *frem2a* coding region from *bla^ta90^* homozygotes, we identified a single 5209C>T mutation that results in the exchange of a strictly conserved arginine residue at amino acid position 1737 by a tryptophan (Figure 3G–3I). This mutation generated a restriction fragment length polymorphism, which we used for direct segregation linkage analysis, revealing co-segregation of the *frem2a* mutation and the *bla* phenotype in 160/160 investigated meioses (Figure 3J). Finally, we identified mutations in the *frem1a* coding region in *rfl* alleles. *rfl^tc280b^* harboured a 1491T>A mutation in the cDNA resulting in the conversion of the triplet encoding tyrosine 497 to a stop codon (Figure 4G and 4H). Similarly, the *rfl^fr23^* allele displayed a 2487T>A mutation leading to a premature stop codon at amino acid position 829 (Figure 4G and 4I), whilst the *frem1a* cDNA sequence from the *rfl^tr240^* mutant fish had a 13 nucleotide insertion corresponding to the last nucleotides of intron 32 of the *frem1a* gene (Figure 4J). This insertion leads to a frame shift, inclusion of 20 amino acids (VSVSDVLQALFSRSLRSPAL) and premature termination of the protein. Consistently, the genomic DNA of *rfl^tr240^* mutants displayed a T>A mutation in intron 32, 15 base pairs upstream of the junction with exon 33, generating a novel and preferentially used splice acceptor site (Figure 4K and 4L).

We could reproduce the *pif, bla* and *rfl* fin blister phenotypes in wild-type embryos by MO-mediated knock-down of *fras1, frem2a* or *frem1a*. The defects of *fras1, frem2a* and *frem1a* morphants were indistinguishable from those of the *pif, bla* and *rfl* mutants, respectively (compare Figure 2C and 2B, Figure 3C and 3B, and Figure 4C and 4B). We also confirmed the *fras1* and *frem1a* splice MO results using second MOs targeting the translation start sites of these genes (Figure S4A and S4B). Together, this indicates that the zebrafish homologues of the human disease genes Fras1, Frem2 and Frem1 are indispensable during zebrafish fin development.

Mouse Fras1 and Frem2 have been shown to interact in vitro and are suggested to reciprocally stabilise each other within the basement membrane [23]. Consistent with this, we found that co-injection of suboptimal doses of *fras1* MO and *frem2a* MO, which upon single injections did not cause apparent defects, yielded severe blistering of the fins comparable to that of *pif* mutants or embryos injected with highest MO amounts (Figure S3E, S3F, S3G, and S3H; Table 2). This synergistic enhancement of defects caused by partial loss of each of the two players is in line with a cooperation of Fras1 and Frem2a during normal fin development.

**Table 2:** Synergistic interaction between *frem2a* and *fras1*.

	uninjected control		*frem2a* ATG MO 1∶60		*fras1* ATG MO 1∶4		*frem2a* ATG MO 1∶60 + *fras1* ATG MO 1∶4	
	C	S	C	S	C	S	C	S
WT fin (%)	100	100	94	98	96	80	33	42
single blister (%)	0	0	4	0	4	20	16	16
multiple blisters (%)	0	0	0	0	0	0	32	21
degenerate fins (%)	0	0	2	2	0	0	19	21
n	79	91	51	101	57	61	109	66

For more information, see Table 1.

### Antisense-mediated inactivation of zebrafish Frem1b, Frem2b, and Frem3 proteins reveals partial functional overlap of zebrafish Fraser complex paralogues

**Figure 5:**
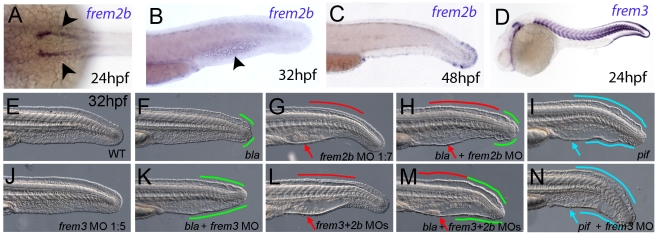
Partially redundant roles of zebrafish Frem2/3 paralogues. In situ hybridisation of embryos using probes against *frem2b* (A–C), and *frem3* (D) at 24 hpf (A, D), 32 hpf (B), and 48 hpf (C). Expression of *frem3* in the fin fold can be seen at 24 hpf (D), whilst expression at this site only commences at 48 hpf for *frem2b* (C). *frem2b* is also expressed in the blood islands at 32hpf (arrowhead, G) and in the pronephric ducts from 24 hpf (arrowheads in A; dorsal view). (E–N) Lateral views of 32 hpf WT embryos (E,G,J,L), *bla^ta90/ta90^* embryos (F,H,K,M) or *pif^tm95/tm95^* embryos (I,N) which are either uninjected (E,F,I), or injected with morpholinos targeting *frem2b* (G,H), *frem3* (J,K,N) or a mix of morpholinos against *frem2b* and *frem3* (L,M). Blistered regions of the fin and blood islands are highlighted by bars and arrows respectively which are coloured green in *bla* mutants, red in *frem2b* morphants and blue in *pif* mutants.

As described above, the blistering phenotypes of *frem1a* (*rfl*) and *frem2a* (*bla*) mutants are significantly weaker or become apparent significantly later than those of *fras1* (*pif*) mutants, suggesting partial functional redundancy among Frem1 and Frem2 proteins. Performing BLAST searches of different zebrafish databases, we identified 3 further members of the Fras/Frem family, which, according to our own phylogenetic analyses and recently published data by the Smyth laboratory [24], have been named *frem1b*, *frem2b* and *frem3*, whereas no second *fras1* paralogue could be identified. At 24 hpf, strong fin fold expression similar to that of *fras1* and *frem2a* was evident for *frem3* (Figure 5D), whereas *frem2b* expression in the fin fold could only be detected starting at the second day of development (Figure 5C). Expression of *frem2b* was also noted in the pronephric ducts at 24 hpf (Figure 5A), as well as the blood islands at 32 hpf (Figure 5B). Expression of *frem1b* in the fin folds was comparably weak and diffuse, while more prominent expression was noted in the blood islands at 24 hpf (Figure 4E) and in the developing vasculature of the head and in the intersomitic boundaries at 5 dpf (data not shown). To understand if these genes also play a function in maintaining fin morphology, we designed MOs against them. Whilst injection of either a splice or ATG MO targeting *frem1b* into WT embryos did not elicit a discernable phenotype, when injected into *rfl^fr23/fr23^* (*frem1a*) mutant embryos, both of these MOs enhanced the blistering phenotype significantly, with blisters also appearing much earlier (at 32 hpf; Figure 4M, 4N–4O, Figure S4C). These blisters seemed quite unstable, and in many cases collapsed by 48 hpf to give the fin a dysmorphic appearance (compare Figure 4P with Figure 4A–4C).

Injection of MOs targeting the translation start site of *frem2b* and *frem3* revealed both functional redundancy with *frem2a* and regional sub-functionalisation. We noted that while *bla^ta90/ta90^* (*frem2a*) mutants displayed small blisters restricted to the posterior medial fin at 32 hpf (Figure 5F), *frem2b* morphants had large blistering in the blood island region as well as in the dorsal region, anterior to the tail tip, sites unaffected in *bla* mutants (Figure 5G; confirmed with an independent 5′UTR directed MO, Figure S4D). Injecting the *frem2b* MO into *bla^ta90/ta90^* embryos had an additive effect, with larvae showing small blisters at the tail tip and blisters in the blood islands and dorsal regions (Figure 5H). In contrast to the *frem2b* MO, injection of the *frem3* MO alone did not yield an appreciable phenotype (Figure 5E and 5J), despite high *frem3* expression in the fin fold. We hypothesised that the function of Frem3 may be redundant with other Fraser genes expressed in the fin fold. However knockdown of *frem3* in either *frem2b* morphants or *pif* mutants failed to enhance their respective phenotypes appreciably (compare Figure 5L and 5G, and Figure 5N and 5I). In contrast, knockdown of *frem3* in *bla^ta90/ta90^* embryos with either an ATG or splice MO, visibly enhanced the severity of the *bla* fin blisters, with anterior expansion of the blistered region (compare Figure 5K and Figure S4E with Figure 5F), but generally without significant blistering of the blood island region (Figure 5K). Finally, triple abrogation of both *frem2* paralogues and *frem3* resulted in embryos phenotypically indistinguishable from *pif* mutants (compare Figure 5M and 5I), consistent with the identical phenotypes of the mouse *Fras1* and *Frem2* mutants. However, embryos deficient in Fras1, Frem2a, Frem2b and Frem3, were no more severely affected than either *pif* mutants alone or the Frem2a, Frem2b and Frem3 triple deficient embryos (Figure S3Y, S3Z, S3AA, and S3AB). Together, this suggests that Frem1a acts in partial functional redundancy with Frem1b, and Frem2a in partial redundancy with Frem2b and Frem3, partially compensating for each other as interaction partners of Fras1.

### Grip1 and Grip2 have partially redundant roles to avoid fin blister formation in zebrafish

**Figure 6:**
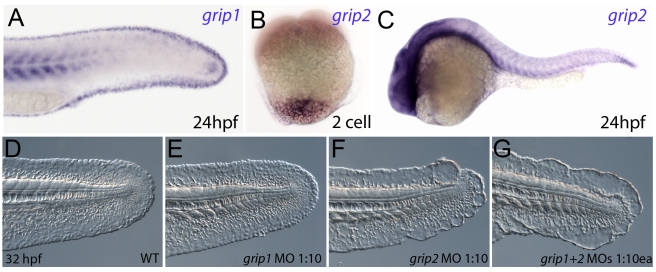
Conserved and redundant roles of zebrafish Grip1/2 proteins. Lateral views of embryos stained by in situ hybridisation with probes against *grip1* (A) and *grip2* (B,C) at 2-cell stage (B) and 24 hpf (A,C). *grip1* is expressed in the fin fold (A) whilst *grip2* is broadly expressed at 24 hpf (C). *grip2* is maternally deposited in a vegetal domain (B). (D–G) Lateral views of an uninjected WT embryo (D) or embryos injected with morpholinos against *grip1* (E), *grip2* (F) or both *grip1 and grip2* (G) at 32 hpf, demonstrating that whilst *grip2* can fully compensate for loss of *grip1* function in fin fold integrity (E), loss of *grip2* alone (F), or combined loss of both proteins (G) generates moderate to severe fin fold blistering respectively.

It has been shown in mouse studies that the intracellular trafficking proteins Grip1 and Grip2 are required for localisation of Fras1 and Frem2 to the basal cell membrane, and that *Grip1, Grip2* double mutant mice resemble *Fras1* mutant mice [9]. We identified zebrafish orthologues of both *Grip1* and *Grip2* and analysed their expression pattern to determine if their role in trafficking Fras1 and Frem2 is conserved. We found that *grip1* was expressed in an identical pattern to both *fras1* and *frem2a*, including the fin fold (Figure 6A), whereas *grip2* displayed rather ubiquitous expression, preceded by maternal transcript localised vegetally, as reported for *Xenopus* X*Grip2* (Figure 6B and 6C) [25]. Upon injection of wild-type embryos with a *grip1* splice MO, we failed to observe any fin phenotype (Figure 6D and 6E). However, injection of MOs targeted to either the ATG or 5′UTR of Grip2 generated mild blistering of the fin (Figure 6F; data not shown). Finally, simultaneous injection of both the Grip1 splice MO and the Grip2 ATG MO produced severe blistering in the fin (Figure 6G), similar to that of *pif* mutants or Frem2a/2b/3 triple deficient embryos. This was confirmed with co-injection of the Grip1 ATG MO and Grip2 5′UTR MOs (Figure S4F). Thus, as in mouse, the Grip proteins display partially redundant functions, and the blistering seen upon their loss points to a conserved role of the Grip1 and 2 proteins in localising Fraser complex proteins in zebrafish.

### FurinA synergistically interacts with Frem2a, and it is required for ectodomain shedding of Fras1 and Frem2 in vitro and for proper Fras1 localization in vivo

**Figure 7:**
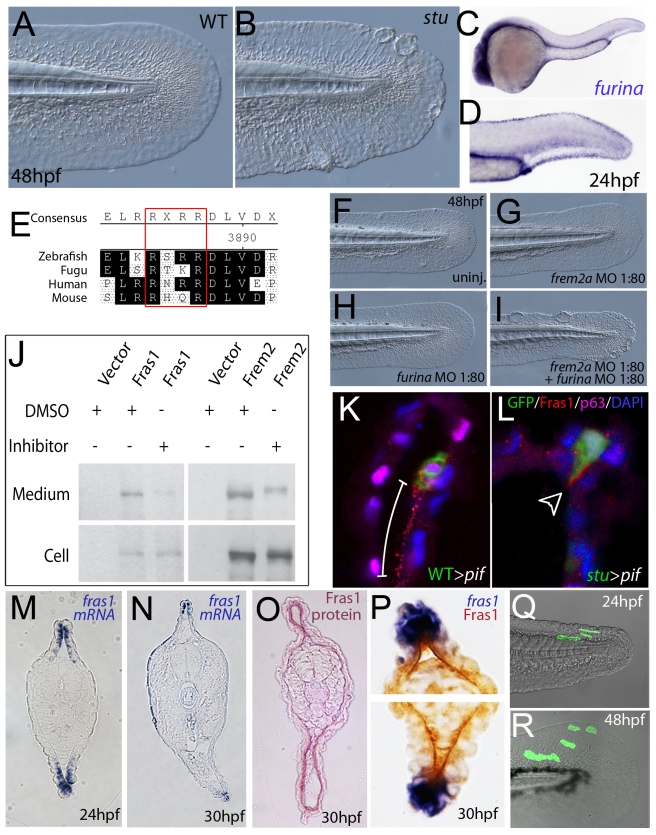
Furin is required for basement membrane anchorage, ectodomain shedding and proper basement membrane localisation of Frem2 and Fras1 proteins. (A,B) Lateral views of the medial fin of a *sturgeon* mutant at 48 hpf (B) showing mild blistering reminiscent of *pinfin* and *blasen* mutants compared to a sibling (A). (C,D) In situ hybridisation analysis of *furina* demonstrates broad expression in the embryo at 24 hpf (C) with clearly increased levels in the fin fold (D). (E) A consensus Furin cleavage site (red box) is conserved in Fras1 immediately N-terminal to the transmembrane domain across vertebrates. (F–I) *furina* and *frem2a* interact dose-dependently in zebrafish. Embryos injected with sub-phenotypic doses of morpholinos targeting *frem2a* (G) or *furina* (H) have medial fins as uninjected control embryos (F) at 48 hpf. Combined injection of the two MOs at these doses robustly induces single or multiple blisters of the fins (I). (J) Chemical inhibition of Furin function (far right lanes in all panels) reduces secretion of N-terminally 3xHA tagged Fras1 protein (upper left panel) and of N-terminally 3xMyc tagged Frem2 protein (upper right panel) from 293F cells into the medium. Proteins were detected by Western Blotting with an anti-HA antibody (for the Fras1 construct) or an anti-myc antibody (for the Frem2 construct). Cellular expression levels were not affected by addition of the inhibitor (lower panels). Due to the large size of the proteins and the small size of the cleaved C-terminus, the differences in sizes between the cellular and secreted proteins are indistinguishable. (K,L) Transverse sections of the posterior medial fin of 42 hpf *pif^te262/te262^* embryos at 42 hpf, after transplantation of GFP-positive cells from either a WT (K) or a *sturgeon* mutant (L) donor at 6 hpf. The sections were immunostained for Fras1 (red), p63 (pink) and GFP (green), and nuclear DNA was counterstained with DAPI (blue). Fras1 protein from WT cells can be found in the basement membrane several cell diameters proximal of its source (white bar in K). In contrast, Fras1 from *stu* mutant cells lacking FurinA remains restricted to the basal surface of the donor cell (arrowhead, L). (M–P) Transverse sections through tail of embryos at 24 hpf (M) or 30 hpf (N–P) showing localisation of *fras1* mRNA by in situ hybridisation (M,N,P: blue precipitate) compared to Fras1 protein (O: red precipitate, P: brown precipitate). Fras1 protein is present in the basement membrane along the entire fin fold (O,P), whereas *fras1* mRNA is largely restricted to apical cells of the fin fold (M,N,P). Apical restriction is more pronounced at 30 hpf (N), while at 24 hpf, the fras1 expression domain appears to extend further proximally (M). (Q,R) Cell tracing analysis after transplantation of GFP-labelled, tg(*bactin*::*hras-egfp*) transgenic presumptive epidermal cells into non-transgenic hosts. Comparison of labelled cells in the same chimeric embryos at 24 hpf (Q) and 48 hpf (R) revealed that proximal clones had approximately doubled their cell number, whilst distal cells had not proliferated, but had increased their surface area by acquiring a flat elongate shape.

The zebrafish craniofacial mutant *sturgeon* (*stu*) in the proprotein convertase FurinA also displays mild blisters in the median fin folds (Figure 7A and 7B) [19], [26]. Consistently, *furina* displayed prominent expression in the apical median fin fold of 24 hpf wild-type embryos (Figure 7C and 7D). Interestingly, Fras1 and Frem2 proteins, in contrast to Frem1, contain C-terminal transmembrane domains. However, recent in vitro studies have shown that both can be shed from the cell membrane, while the proteases potentially mediating this effect remained unknown [23]. We identified conserved Furin consensus cleavage sites in the zebrafish Fras1 and Frem2a protein sequences (Figure 7E). These occurred in both proteins immediately N-terminal to the predicted transmembrane domain. If FurinA does process Fras1 and/or Frem2a to a mature form, we would expect them to interact dose-dependently. We tested this by injecting sub-phenotypic doses of morpholinos against *furina* and *frem2a*, and were indeed able to induce fin blisters when combining doses of the MOs that individually gave no phenotype (Figure 7F, 7H, and 7I; Table 3). We then used a previously reported in vitro biochemical assay [23], which demonstrated that both murine Fras1 and Frem2 are released into the medium of transfected 293F cells. This cell line expresses Furin endogenously (data not shown) (Figure 7J). The relative amount of Fras1 and Frem2 protein in the medium was significantly reduced after addition of the Furin/Proprotein convertase inhibitor Decanoyl-RVKR-CMK, while cellular protein levels remained unaffected (Figure 7J). This indicates that membrane shedding of both proteins is indeed dependent on Furin or a related Proprotein convertase. The similar phenotypes of *fras1, frem2a* and *furina* mutants further suggest that such Furin-dependent ectodomain shedding of Fras1 and Frem2a is essential for their role to ensure proper basement membrane integrity.

**Table 3:** Synergistic interaction between *frem2a* and *furina*.

	uninjected controls		*frem2a* ATG MO 1∶80		*furina* ATG MO 1∶80		*frem2a* ATG MO 1∶80 + *furina* ATG MO 1∶80	
	C	S	C	S	C	S	C	S
WT fin (%)	100	100	100	98	100	73	39	32
single blister (%)	0	0	0	1	0	23	9	14
multiple blisters (%)	0	0	0	0	0	2	40	49
degenerate fins (%)	0	0	0	1	0	2	12	5
n	59	76	43	100	33	95	77	87

Embryos were assessed at 30 hpf. For more information see Table 1.

To further assess the role of Furin in Fras1 shedding in the zebrafish fin, we used a transplantation approach to track the behaviour of the Fras1 protein and its dependence on FurinA in vivo. First, GFP-positive wild-type cells were transplanted into Fras1-deficient *pif* mutant hosts, followed by immunofluorescence stainings on transverse sections through median fins with a polyclonal antibody raised against zebrafish Fras1 (see Materials and Methods). In non-chimeric wild-type embryos, Fras1 protein was present below the epidermal sheets of the median fin (Figure 7O), consistent with the reported localization of mouse Fras1 to basement membranes [23]. Since the *pif^te262/te262^* host cells fail to generate Fras1 (the *pif^te262^* allele is a nonsense mutation N-terminal to the region used to raise the antibody; see also below, Figure 9I), all protein detected by the antibody in wild type > *pif* chimeras must originate from the transplanted, GFP-labelled wild-type cells. Indeed, we only detected anti-Fras1 signals associated with transplanted cells. However, in case of transplanted wild-type cells, Fras1 signals were not restricted to the region of the basement membrane directly underlying the transplanted cells, but found in a significantly larger portion of the basement membrane, extending several cell diameters proximally (but not distally) of the donor cells (Figure 7K; n = 38/41; 4 embryos). This suggests that Fras1 is shed from the surface of the donor cell to undergo some kind of directed unilateral displacement within the basement membrane (see also below). Identical transplantation experiments with Furina-deficient, rather than wild-type donors, further revealed that this displacement requires the donor cell to express FurinA Thus, in contrast to Fras1 from transplanted wild-type cells, Fras1 derived from cells of *stu* mutant donors injected with moderate amounts of *furina* MO did remain closely attached to the basal cell surface, pointing to a lack of shedding (arrowhead in Figure 7L; n = 24/24; 2 embryos). Corresponding shifts in the localisation of Fras1 protein were also observed in non-mosaic *stu* mutants. Whereas in wild-type siblings, Fras1 protein was found in the basement membrane throughout the entire proximo-distal extent of the median fins (Figure S5A; see also below), it was restricted to more distal regions in *stu* mutants (Figure S5B). Together, this suggests that FurinA acts as a Fras1 sheddase, and that this shedding is a prerequisite for the relative proximal-wards displacement of Fras1 protein within the forming median fins.

### The distribution of Fras1 protein within developing basement membranes is broader than the expression domain of the *fras1* gene

Data consistent with such a proximal-wards displacement of Fras1 protein were also obtained when directly comparing the distribution patterns of *fras1* mRNA and Fras1 protein. At 30 hpf Fras1 protein was found along the entire proximo-distal extent of fin (Figure 7O and 7P), consistent with the proximal extension of the fin blisters in mutant embryos (see above). In contrast, *fras1* RNA was strictly confined to the apical-most epidermal cells of the fin folds (Figure 7N and 7P). Aside from the displacement of Fras1 protein relative to the overlying cells mentioned above, a second explanation for this proximally extended distribution of Fras1 protein could be apical growth of the fin fold, whereby descendents of *fras1*-positive distal cells would give rise to more proximal fin fold epithelia, carrying closely associated Fras1 proximally as they generate the proximal fin. To test this notion, we performed in vivo cell tracing experiments with clones of fluorescently labelled ectodermal cells. However, in none of our recorded cases (0/6) did cells located in apical ectodermal ridges at 24 hpf give rise to more proximal fin cells at 48 hpf (Figure 7Q and 7R). Rather, fin extension seemed to be driven by uniform growth along the entire proximo-distal axis of the fin or by preferential proliferation of epidermal cells in more proximal positions. This rules out apical-driven growth as a mechanism for proximally extended Fras1 protein distribution. A third explanation could be dynamics in the *fras1* expression pattern in combination with high Fras1 protein stability. Indeed, we noted that in transverse sections at earlier stages, the *fras1* RNA expression domain extended more proximally than later (compare Figure 7N with Figure 7M). Together, these results suggest that Fras1 protein is distributed in the basement membrane along the entire proximal-distal fin axis, which may be accounted for by high stability of Fras1 protein deriving from the initially broader RNA expression domain, coupled with proximal growth of the fin fold epidermis over basement membrane material deposited by apical cells, and/or directed proximal-wards motility of shed Fras1 protein within the basement membrane.

### The fin blistering of *nagel* mutants is caused by mutations in Hemicentin1 (Hmcn1)

**Figure 8:**
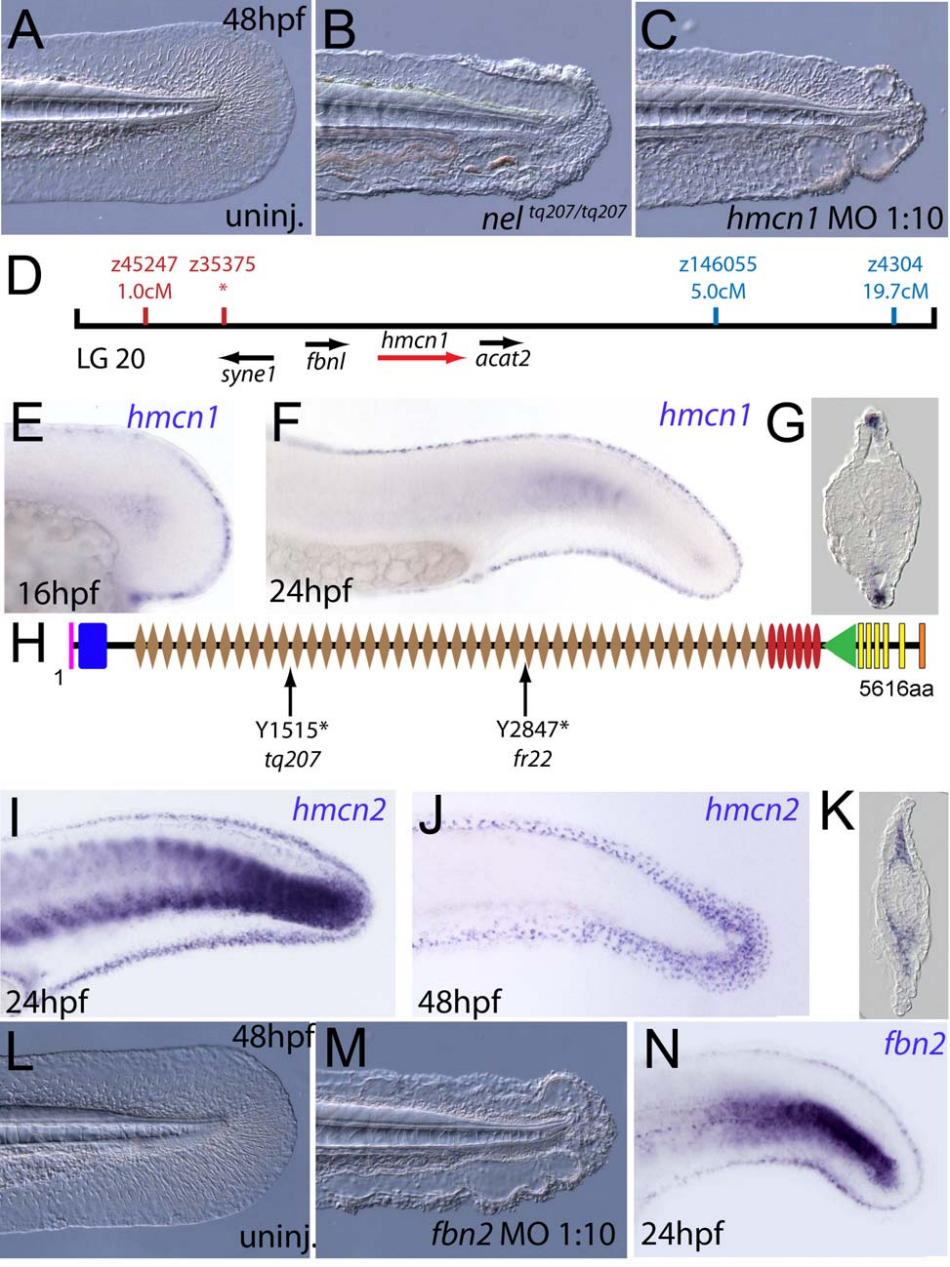
The fin blistering of *nagel* mutants is due to mutations in the *hemicentin1* gene. (A–C) Lateral views of the posterior medial tail fin of WT larvae at 48 hpf injected with a morpholino targeting the translation site of *hmcn1* mRNA (C). Blisters reminiscent of *nagel* mutant embryos (B) are clearly visible compared to age-matched uninjected controls (A). (D) Genetic map showing the location of the *nagel* locus on LG20, which did not recombine with the SSLP marker z35375. The genes within the interval, including *hmcn1* (red arrow), are shown below. (E–G) Lateral views (E,F) and transverse section (G) of embryos stained by in situ hybridisation with an *hmcn1* probe, showing expression in somites (F) and in the apical region of the fin fold at 16 hpf (E), 24 hpf (F), and at 48 hpf (G). (H) The zebrafish Hmcn1 protein is 5616 amino acids in length and contains a signal peptide (pink bar), a Von Willebrand factorA domain (blue box), 44 Ig-like domains (brown diamonds), six thrombospondin type-1 repeats (red ovals), a nidogen G2 domain (green triangle), five EGF-like domains (yellow bars) and a Neuralized homology repeat domain (orange bar). Mutations of the two sequenced *nagel* alleles and their relative locations are indicated. (I–K) Lateral views (I,J) and transverse section (K) of embryos stained by in situ hybridisation showing expression of *hmcn2* in the tail region of the zebrafish embryo at 24 hpf (I) and 48 hpf (J,K). While at 24 hpf, *hmcn2* is expressed in the fin fold epidermis and the somites, (I), it becomes confined to fin mesenchyme cells at 48 hpf (J,K). probe Lateral view of a 48 hpf embryo injected with *fibrillin2* MO, showing strong blistering of the medial fin (M) compared to uninjected control (L). (N) Lateral view showing expression of *fbn2* in the medial fin fold of the tail at 24 hpf as well as in floor plate, hypochord and notochord.

We next turned our attention to the last fin blister mutant, *nagel* (*nel*; Figure 8A and 8B). Despite showing strong blistering, with onset at a similar time to *pif* and *bla*, *nel* appears slightly weaker than *pif* and only occasionally shows blisters in the blood islands (Figure S1F, S1N and S1V). We mapped the *nel^tq207^* mutation to LG20, close to marker z35375, but distant from all annotated *fras*/*frem*/*grip* genes (Figure 8D). One of the genes located within the interval was *hemicentin1* (*hmcn1*; Genbank accession number GU936666), which encodes a large multidomain ECM protein of the Fibulin family, the function of which has thus far solely been investigated in the nematode *C. elegans*. In this organism, Hemicentin is required for proper attachment of cells to the epidermis and for basement membrane organisation in the gonads [27], [28]. Whole mount in situ hybridisations revealed that zebrafish *hmcn1* was expressed in the apical median fin fold epithelium from 20 hpf onwards (Figure 8E–8G), similar to the expression patterns of *fras1* and *frem2a*. Consistent with a role in fin fold development, injection of a translation-blocking *hmcn1* MO generated embryos with fin blisters, resembling *nel* mutants (Figure 8B and 8C). Furthermore, we found nonsense mutations in the *hmcn1* coding region of both sequenced *nel* alleles (Figure 8H). The *nel^tq207^* allele displayed a 4545C>G substitution, which leads to a premature termination of Hmcn1 after 1514 of 5616 amino acid residues, whilst the *nel^fr22^* allele contained a nonsense mutation and an adjacent splice donor site-creating mutation, both of which cause a C-terminal truncation of Hmcn1 after half of the protein (for details, see Figure S6A, S6B, S6C, S6D, S6E, S6F, and S6G). Together, these data indicate that the fin blistering of *nel* mutants is caused by loss-of-function mutations in the *hmcn1* gene.

We also identified zebrafish *hmcn2* (Genbank accession numbers GU936667 and GU936668), a second *hemicentin* paralogue also present in mammals [29]. In contrast to the restricted expression of *hmcn1* in epithelial cells of the apical fin fold, *hmcn2* transcripts were present both in the fin fold epithelium and the fin mesenchyme at 24 hpf (Figure 8I), and restricted to the fin mesenchyme at 48 hpf (Figure 8J and 8K). However, neither a translation-blocking, nor a splicing-blocking *hmcn2* MO yielded a consistent phenotype alone, nor did the *hmcn2* MO clearly enhance the *nel* phenotype (data not shown). This leaves the role of Hmcn2 during zebrafish development currently unclear.

### Hemicentin1 synergistically interacts with Fibrillin-2 and Fras1, but not with Lamininα5

During a morpholino screen of genes up-regulated in muscle fibres, we observed fin blistering in embryos injected with an MO against *fibrillin2* (*fbn2*), similar to that of *fras1* and *hmcn1* mutants (Figure 8L and 8M). Indeed this was confirmed by the recent report of a zebrafish *fibrillin2* mutant, *puff daddy* (*pfd^gw1^*), isolated in an ENU screen and characterised by defects in notochord and vascular morphogenesis, but also displaying blistering of the fin fold [18]. Furthermore, like *fras1* and *hmcn1*, *fbn2* displayed expression in the median fin fold epithelium (Figure 8N). Fibrillin-1 has been shown to directly interact with members of the Fibulin protein family [30], [31]. Given the similarity of phenotype, we hypothesised that Hmcn1 (also called Fibulin-6) and Fbn2 might similarly interact during zebrafish fin development in vivo, and carried out synergistic enhancement studies, as described above for *fras1* and *frem2a*. Indeed, while individually, neither the *hmcn1* nor the *fbn2* MO elicited a phenotype at low doses, when combined, they generated fin blisters as in *hmcn1* mutants (Figure S3I, S3J, S3K, and S3L; Table 4). Thus Fibrillin2 and Hemicentin1 appear to act in concert to maintain fin fold structure. Curiously, injection of strong doses of *fbn2* MO into *nel^tq207/tq207^* mutants realised embryos with fin blistering much stronger than in either *nel* mutants or strong *fbn2* morphants alone (Figure S3AC, S3AD, and S3AF). However, resulting embryos were indistinguishable from *pif* mutants or *frem2a/2b/3* triple morphants (Figure S3Y, S3Z, S3AA, and S3AB). This suggests Hmcn1 and Fbn2 can partially compensate for each other and highlights the complex interplay of ECM molecules maintaining fin fold integrity.

**Table 4:** Synergistic interaction between *fbn2* and *hmcn1*.

	uninjected control		*fbn2* ATG MO 1∶40		*hmcn1* ATG MO 1∶20		*fbn2* ATG MO 1∶40 + *hmcn1* ATG MO 1∶20	
	C	S	C	S	C	S	C	S
WT fin (%)	100	100	44	97	96	100	49	46
single blister (%)	0	0	0	3	2	0	2	13
multiple blisters (%)	0	0	0	0	0	0	49	41
degenerate fins (%)	0	0	0	0	2	0	0	0
n	59	84	44	74	47	62	39	74

For more information see Table 1.

The synergistic interaction between Fras1 and Frem2 on one side and Hmcn1 and Fbn2 on the other side is consistent with previous biochemical reports on these or other family members. To investigate whether the two ECM complexes also cooperate with each other, which has not been reported as yet, we next carried out synergistic interaction studies between Hmcn1 and Frem2a/Fras1. To study embryos completely lacking both Hmcn1 and Fras1 function, we generated *pif^te262/te262^*; *nel^tq207/tq207^* double mutants. Double mutants were as strong as *pif* single mutants (Figure S3U, S3V, and S3X). However, combined partial loss of Hmcn1 and Fras1 had a synergistically enhancing effect (Figure S3M, S3N, S3O, and S3P; Table 5), similar to the effect between Fras1 and Frem2a (Figure S3E, S3F, S3G, and S3H; Table 2). In contrast, combined injections of sub-phenotypic doses of *hmcn1* and *lama5* MOs, although effective in dose-dependent interaction studies with *frem2a* or *itga3*, respectively (Table 1), failed to produce blistering or dysmorphic fins (Figure S3Q, S3R, S3S, and S3T; Table 6). Together, this points to a common role of Fras1/Frem2a/Hmcn1/Fbl2 in the basement membrane of developing fin folds, which is distinct from that of Lamα5/Itgα3 complexes.

**Table 5:** Synergistic interaction between *fras1* and *hmcn1*.

	uninjected control		*fras1* ATG MO 1∶10		*hmcn1* ATG MO 1∶20		*fras1* ATG MO 1∶10 + *hmcn1* ATG MO 1∶20	
	C	S	C	S	C	S	C	S
WT fin (%)	47	127	46	151	90	100	36	59
single blister (%)	0	0	0	0	6	0	21	8
multiple blisters (%)	0	0	1	0	4	0	41	32
degenerate fins (%)	0	0	0	0	0	0	2	1
n	47	127	47	151	49	111	104	121

For more information see Table 1.

**Table 6:** No synergistic interaction between *lama5* and *hmcn1*.

	uninjected control		*lama5* splice MO 1∶80		*hmcn1* ATG MO 1∶20		*lama5* splice MO 1∶80 + *hmcn1* ATG MO 1∶20	
	C	S	C	S	C	S	C	S
WT fin (%)	100	100	100	99	91	100	98	100
single blister (%)	0	0	0	0	7	0	2	0
multiple blisters (%)	0	0	0	0	2	0	0	0
degenerate fins (%)	0	0	0	1	0	0	0	0
n	42	143	51	117	45	113	49	161

For more information see Table 1.

### Fin blistering of *fras1* and *hmcn1* mutants occurs at the level of the sublamina densa

**Figure 9:**
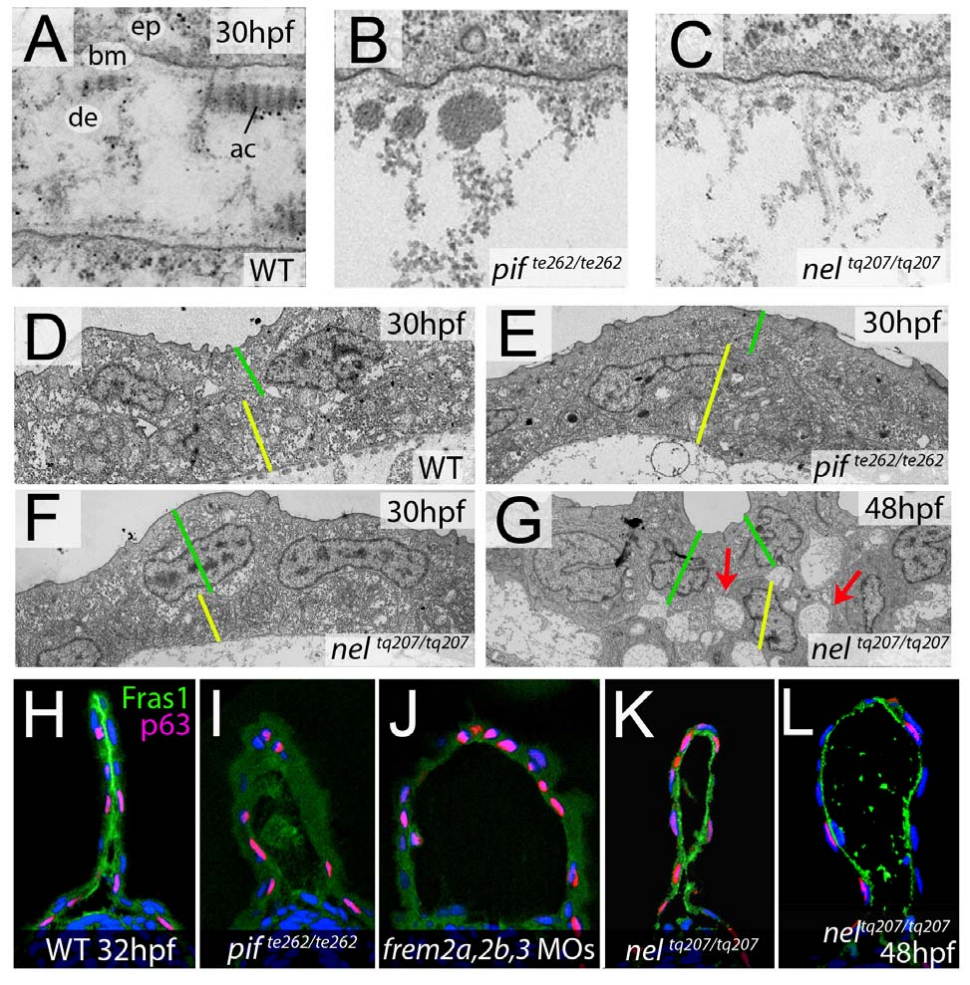
*fras1* and *hmcn1* mutants display blister formation at identical sites below the basement membrane; however, in contrast to Frem2/3 proteins, Hmcn1 is dispensable for Fras1 stabilisation. (A–C) Electron micrographs of the sub-epidermal space of medial fin folds at 30 hpf in WT (A), *pif^te262/te262^* (B), and *nel^tq207/tq207^* (C) embryos. Epidermal cells (indicated by ‘ep’ in A) are at the top of all panels as well as in the lower part of A. The lamina densa of the basement membrane (labelled with ‘bm’ in A), including the sublamina densa, is attached to the epidermal cells in all three panels. Below the bm, electron dense material is evident as actinotrichia (ac) or dermis material (de), which appears disorganized and in the two mutants (B,C). (D–G) Low power electron micrographs of the apical portion of the fin fold in WT (D), *pif^te262/te262^* (E), and *nel^tq207/tq207^* (F–G) embryos at 30 hpf (D–F) and 48 hpf (G). Cell–cell adhesion is well preserved in both mutants at 30 hpf (E–F), but at 48 hpf, when the blister begins to collapse, cell-cell boundaries become compromised (G, red arrows). The basal and outer enveloping layers are indicated with yellow and green bars respectively. (H–L) Transverse sections fluorescently immunostained for Fras1 (green), epidermal p63 (red) and counterstained with DAPI (blue) at 30 hpf (H–K) and 48 hpf (L). Fras1 immunoreactivity is evident between the fin folds in WT embryos (H), but lost in *pif^te262/te262^* mutants (I) as well as in embryos injected with MOs against *frem2a*, *frem2b*, and *frem3* (J). In contrast, it is retained in *nel^tq207/tq207^* embryos at both 30 hpf (K) and 48 hpf (L), when blistering is even more severe.

In mouse Fras1 and Frem mutants, embryonic skin blistering occurs at the level of the sublamina densa of the basement membrane, with the BM remaining attached to the basal cell surface overlying the blister cavity [23], [32]. Transmission electron microscopy studies revealed that the same is true for the median fin blisters of the zebrafish *pif* (*fras1*) and *nel* (*hmcn1*) mutants, with the blister cavity forming below the lamina densa, at the interphase of the basement membrane and the underlying dermis (Figure 9A–9C). This indicates that zebrafish and mammalian Fras1 play comparable structural roles within developing basement membranes anchorage within the embryonic skin. Furthermore, it suggests that Fras1 and Hmcn1 most likely act at the same sites within basement membranes, in line with the aforementioned synergistic interaction between the two genes.

### Cell–cell adhesion is initially unaffected in blister mutants

Previous electron microscopy studies of zebrafish *lama5* mutants have indicated defects in both epidermis – basement membrane association as well as in epidermal cell-cell adhesion [21]. We analysed the electron micrographs to establish if cell-cell adhesion was also affected in the *pif^te262/te262^* and *nel^q207/tq207^* blister mutants. It appeared that at 30 hpf, cells in the epidermis of the fin maintained good adhesion with neighbouring cells despite having detached from the dermis (Figure 9D–9F). This is in line with the stable nature of the blisters at this stage. However by 48 hpf, the fin blisters are beginning to collapse as the fin fold grows, and the fins show signs of dysmorphogenesis. Ultrastructurally, large cavities can be seen between basal cells and between basal cells and overlying enveloping layer (EVL) cells (Figure 9G, red arrows). Thus, it appears that initially cell-cell contacts are not affected by the blistering below the basement membrane, whereas later cell-cell adhesion defects can be seen concomitant with the onset of overall fin degeneration.

### Hmcn1 does not affect the stability or distribution of Fras1 protein

Mouse Fras1 and Frem2 proteins have been shown to physically bind to and stabilise each other [23], possibly accounting for the observed genetic synergism between *fras1* and *frem2a* in zebrafish described above. To study whether a similar biochemical interaction might also apply to zebrafish Fras1 and Frem2 proteins, and whether Fras1 stability might in addition require Hmcn1, accounting for the revealed genetic synergism between *fras1* and *hmcn1*, we performed Fras1 immunostainings in *pif* mutants, *frem2a/b/3* morphants and *nel* mutants. Whilst we observed strong Fras1 immunostaining within the fin fold of wild-type embryos at 32 hpf (Figure 9H), immunostaining was absent both in *pif^te262/te262^* mutants (Figure 9I; compare with Figure 7K and 7L) and in embryos deficient for *frem2a, frem2b* and *frem3* (Figure 9J), consistent with the reciprocal stabilisation of these proteins. In contrast, we observed clear Fras1 immunostaining, basal to the epidermal cells and at the lateral edges of both nascent and older blisters of *nel^tq207/tq207^* mutants (Figure 9K and 9L). This demonstrates that in contrast to Frem2, Fras1 stabilisation does not require Hmcn1.

## Discussion

Studying the processes involved in medial and paired fin development of lower vertebrates has implications for understanding the aetiology of human limb malformations [33], [34]. There are a large number of syndromic limb malformations reported [reviewed in 35]. Included in these is Fraser Syndrome, which presents a broad range of defects including cutaneous syndactyly of the limbs. Based on analysis of the mouse ‘bleb’ mutants which model Fraser syndrome, this syndactyly is hypothesised to be a consequence of blistering of the apical ectodermal ridge of the developing limbs. Two of the disease genes underlying Fraser Syndrome in humans were identified as FRAS1 or FREM2, which encode structurally related basement membrane proteins. Mutations in FRAS1 or FREM2 were found in approximately 50% of investigated cases of Fraser Syndrome, whereas the molecular lesions underlying the other half remain unknown.

### The known bleb mutant genes *Fras1*, *Frem1/2*, and *Grip1/2* have conserved roles in zebrafish

We have cloned zebrafish mutants with embryonic blistering of both the medial fin fold and the paired fins. Two of the loci, *pinfin* (*pif*) and *blasen* (*bla*), map to and have lesions in the *fras1* and *frem2a* genes, thus demonstrating that these mutants represent zebrafish models of Fraser Syndrome. We have further confirmed this by reproducing the phenotypes by antisense morpholino knockdown of these genes, however, due to the large size of their genes and mRNAs, rescue experiments with either BACs or in vitro synthesized mRNAs were impossible. Nonetheless our data clearly demonstrate that mutations in Fras1 and Frem2 related proteins in zebrafish yield blistering of the apical ectodermal ridges analogous to that occurring in mammalian mutants for these genes. Similar blistering is seen the zebrafish *rafels* mutants, which we have identified as harbouring mutations in the *frem1a* gene, an orthologue of mouse *Frem1*. Mouse Frem1 mutants (*head blebs*) also belong to the ‘bleb’ class of mutants, exhibiting embryonic blistering of the extremities although with background variability. Whilst the phenotype of *rafels* further extends the homology of the role of the Fraser complex proteins in AER morphogenesis, it is noteworthy that a recent report has described human patients bearing *FREM1* mutations which display bifid nose and anorectal malformations but not the classic Fraser syndactyly, cryptophthalmos or ablepharon, although they do show renal agenesis similar to the Fraser syndrome patients [36]. This highlights the proposal that Frem1 plays a slightly different function to Fras1/Frem2, contrasting the largely indistinguishable phenotypes obtained upon loss of Fras1, Frem2 or Frem1 function in mouse and zebrafish.

In zebrafish, we found *frem1a* to display a partially redundant role with its paralogue *frem1b*, and *frem2b* to display a partially redundant role with *frem2b* and *frem3*. Whilst it appears that both *frem*2b and *frem3* are expressed, to varying extents, in the fin folds at some stage, only loss of Frem2b generated strong fin blistering when injected alone, presenting mostly in the blood island region of the ventral medial fin. Interestingly this site is largely unaffected in the *frem2a* mutant embryos, suggesting regional sub-functionalisation of the Frem2 role between the two paralogues. Finally, we show that antisense knockdown of *frem3*, which does not generate a phenotype by itself, strongly enhanced the fin blistering of *frem2a* mutants (or morphants), whereas it had no effect in the *frem2b* morphant or *fras1* mutant background. We also noted that the loss of both Frem2 proteins and Frem3 resulted in blistering of the same severity as *pif (fras1)* mutants. Together, this indicates partial functional redundancy between Frem2 and Frem3 proteins. Indeed, zebrafish Frem3 appears to have identical domain structure to the Frem2 proteins. This is the first loss-of-function analysis for Frem3 in any organism, since in contrast to Frem1 and 2, no mouse Frem3 mutant has been reported as yet.

One further family of genes contributing to the Fraser protein complex, are the intracellular PDZ domain containing proteins Grip1 and Grip2. These have both been shown to interact with the conserved C-terminal residues of Frem2 and Fras1 and localise them correctly to the basal side of the epidermal cell, from where they can be secreted into the basement membrane [9]. We have shown that zebrafish *grip1* is expressed in an overlapping fashion to the *fras/frem* genes and that depleting the protein levels of both *grip1* and the maternally and ubiquitously expressed *grip2*, realised strong fin blistering. Thus we have demonstrated that all known genes contributing to the human Fraser Syndrome or the mouse ‘bleb’ phenotype generate fin blisters in the zebrafish and conclude that the zebrafish is a valid model for Fraser Syndrome.

Fraser syndrome is a complex disease and presents with multiple pleiotropic defects, all of which seem to derive from spatially restricted and transient basement membrane disruption. Aside from the limb abnormalities, patients sometimes also display renal agenesis, craniofacial dysmorphism, and cryptophthalmos or ablepharon, however there are numerous other defects reported. There is significant clinical variability and no single phenotype is always present [1]. Of the other major diagnostic criteria of Fraser Syndrome, we have only noted craniofacial defect (unpublished data). Intriguingly we have not found any evidence of renal cysts or malformations, which, however, may be due to a lack of ureteric branching in zebrafish – the kidney of zebrafish larvae consists of a single nephron.

### Fras1 and Frem2 might be released from the cell surface via proteolytic cleavage by Furin proprotein convertases

Fras1 and Frem2 contain a C-terminal transmembrane domain. However, according to recent data obtained in cell culture studies, they can be shed from the cell surface. The proteases mediating such ectodomain shedding remained unidentified [23]. Here, we provide both genetic and biochemical evidence that in zebrafish, the proprotein convertase FurinA is involved, and that Furin-mediated ectodomain shedding is important for proper function of Fras1 and/or Frem2 within the fin fold basement membrane (Figure 7). As direct in vivo evidence for this notion, we have studied the localisation behaviour of Fras1 protein in chimeric embryos and in the presence or absence of FurinA (Figure 7K and 7L). We observed that in a wild-type environment, Fras1 protein can indeed be found in the basement membrane distant from its source cell, showing that it does not remain membrane tethered in vivo. Rather, it seems to be shed, allowing the protein to move relative to the overlying cell. By mechanisms we do not fully understand as yet, but which might involve the observed higher proliferation rates of epidermal cells in proximal positions of the forming fins (Figure 7Q and 7R), this Fras1 displacement seems to be directed, occurring in a distal-to-proximal direction only, but not vice versa. Critically, we were able to show that FurinA is required for this Fras1 displacement, as Fras1 was retained on the baso-lateral surface of transplanted *furinA* (*stu*) mutant cells. This also shows that FurinA fulfils it indispensable sheddase role in a cell-autonomous manner within the Fras1-generating cells itself. This is consistent with recent results, demonstrating Furin-mediated shedding of transmembrane collagens like Collagen XXIII in the Golgi network, but not at the cell surface, of cultured keratinocytes [37].

We noted, however, that the blistering seen in *sturgeon* (*stu*; *furina*) mutants was less penetrant than in the *pinfin* or *blasen* mutants. Additionally, failed Fras1 protein displacement was only observed for *stu* mutant cells in rather posterior positions of the median fin, but not in more anterior positions (data not shown), consistent with the location of the blisters in *stu* mutants. We attribute this to regional-specific differential redundancy of FurinA with other Fras1 sheddases, or to regional- or temporal-specific compensation by maternally supplied *furina* transcripts, which are not affected in *sturgeon* mutants. We attempted to fully abolish maternal compensation by use of a morpholino against the translation start site of *furina*, however, this generated strongly dorsalised cells or embryos lacking all posterior structures (TJC and MH, unpublished observations), presumably due to failed processing of Bone Morphogenetic Proteins (BMPs), known targets of Furins which are implicated in early dorsoventral patterning of the zebrafish embryo [38]. The presence of a second *furin* orthologue in the zebrafish (*furinb*) combined with yet other related proprotein convertases, might also partly compensate for the loss of zygotically generated FurinA protein in *sturgeon* mutants. In reverse, FurinA might have other target proteins in addition to Fras1 and Frem2. Thus, we also noted mildly compromised fin morphogenesis and a ruffled appearance of the fins of *sturgeon* mutants. This is likely to be the result of failed processing of other known targets of Furin involved in fin morphogenesis, such as Itga3 [39] and collagens [37]. In conclusion, our data point to a novel role of a Furin proprotein convertase in fin development and the formation of a functional Fraser complex to allow proper basement membrane anchorage.

### Hemicentins, Fibrillin2, and the Fraser complex

The third fin blistering locus we positionally cloned was *nagel* (*nel*), which we found to encode Hemicentin1 (Hmcn1), like Fras1 and the Frem proteins another potential basement membrane protein (Figure 8). As *nel* represented one of the highest hit loci in the original Tubingen mutagenesis screen [40], we reasoned that the gene was likely to encode either a very large protein or a very well conserved protein (thus sensitive to substitution mutations), both of which is the case. While nothing was known about Hemicentin1 function in vertebrates, the *C. elegans* orthologue has been shown to be involved in organising epithelia attachment [27]. We identified two *hmcn1* nonsense mutations in *nel* alleles and thus describe the first *hemicentin* mutant in a vertebrate species. We further showed that whilst *hmcn1* and *fras1* synergistically interact in the fin fold, the presence of Fras1 protein was unaffected in *nel* mutants. This is in contrast to the indispensable effect of Frem2 on Fras1 stability (Figure 9), consistent with the reciprocal stabilisation of mammalian Fras/Frem proteins in the basement membrane [23]. In conclusion, in contrast to Frem proteins, Hmcn1 does not seem to be required for Fras1 stability. Hmcn1 antibodies need to be raised to investigate whether conversly, Fras1 is also dispensable for Hmcn1 stability. In *C. elegans*, Hemicentin is associated with hemidesmosome-type structures, mediating attachment between epithelial cells and the underlying basement membrane. However this is not necessarily true in zebrafish, which does not generate visible hemidesmosomes until 3dpf [41], well after the first observable *nagel* phenotype. Rather, according to our EM studies, Hmcn1 is required for proper attachment of the basement membrane to the underlying dermal compartment. Furthermore, the phenotypes of *nel* and *pif* mutants at both the morphological and ultrastructural level, combined with the synergistic interaction studies, strongly points to a previously unrecognised requirement for Hmcn1 in generating a fully functional Fraser complex.

Curiously, unlike the nonsense *fras1* alleles, which die between 11–12 dpf, the *hmcn1* alleles are adult viable and do not display any overt phenotype, pointing to differential dependence of the Fraser complex on Hmcn1 in different organ contexts. The reason for the larval death of strong *pif* mutants is currently unclear, however the mutant larvae fail to inflate a swim bladder, and remain at the bottom of the tanks lying on their sides, unable to feed. We have shown that in addition to the fin folds, *fras1* is expressed in the brain (midbrain-hindbrain boundary/cerebellum), the ear and the craniofacial system. In addition to the fin blistering, *pif* mutants display subtle craniofacial defects (J. Coffin Talbot et al., unpublished data), and we propose that the observed compromised swimming behaviour of mutants might be due to neurological and balance defects, altogether resembling the craniofacial, ear and neurological phenotypes that are diagnostic criteria for human Fraser syndrome [1]. However, more detailed investigation beyond the scope of this work is required to fully understand these later phenotypic traits.

For the embryonic fin blistering mutants that survive, we noted that generally there is no overt adult fin phenotype, with the exception of the *pif^tm95b^* mutants. There could be two explanations for this. Firstly, during later developmental stages, the described partial functional redundancy, e.g. between *frem1a*/*1b*, or between *frem2a*/*2b*/*3* might become even more prominent. Indeed, most of them are co-expressed in adult fins (data not shown). Alternatively, as demonstrated in the mouse, the Fraser complex in its entirety might only have a transient requirement during embryogenesis, whereas later, its function in tethering the BM to the underlying dermis is taken over by Collagen VII [12]. Of all viable blistering mutants, only the weak *fras1* allele, *pif^tm95b^*, showed a reduced and mis-patterned adult fin. Whilst this could reflect the lack of a paralogous gene to compensate for its function (the zebrafish genome appears to contain only one *fras1* gene), it may also be due the dominant nature of this mutation, with potential disruption to other basement membrane or dermal components during adult fin morphogenesis. Identification and analysis of other mild viable *pif* alleles should help to resolve this point.

*hemicentin2* (*hmcn2*) is also expressed in the fin fold during embryogenesis, however, mostly in the fin mesenchyme. The role of this cell population during fin morphogenesis is presently unknown and we sought to determine the function of *hmcn2* through morpholino knockdown. However, injection of a translation-blocking MO led to no observable fin phenotype, even when injected into *hmcn1* mutants, leaving the function of Hmcn2 unclear.

The Hemicentins belong to the Fibulin family of proteins, characterized by the presence of a C-terminal Fibulin domain. Other members of the Fibulin family (2,4,5) are known to directly bind Fibrillin-1, which is involved in elastic microfibril formation [30]. We found zebrafish *fibrillin2* (*fbn2*) to be co-expressed with *hmcn1* and the *fras1/frem2* genes in the apical fin fold epidermis, while morpholino-based *fbn2* knockdown generated fin blistering phenotype comparable to that of *nel* and *pif* mutants (Figure 8). This phenotype has been confirmed in the *fbn2* mutant *puff daddy* [18]. Furthermore, we could demonstrate a dose-dependent interaction between zebrafish Hmcn1 (also known as Fibulin-6; see above) and Fbn2, thereby extending the known associations between Fibrillins and Fibulin-type proteins, and revealing that Hmcn1 and Fbn2 cooperate to mediate epidermis-basement membrane and/or basement membrane-dermis attachment in vivo. One implication from our work is that the Fraser complex is linked to fibrillin-containing microfibrils within the dermis via Hemicentin1. We are currently applying biochemical approaches to test this notion.

Consistent with an involvement of Fbn2 in Fraser complex function, Fbn2-deficient mice display limb defects ranging from cutaneous to skeletal syndactyly, reminiscent of the ‘bleb’ mutant mice [42]. The embryonic phenotype in *Fbn2^−/−^* null mice has not been reported, however, it is tempting to predict that there may be transient distal limb blistering.

For the future, it will be interesting to characterise the function of Hemicentins in mammals, in particular generating and analyzing mouse mutants lacking Hmcn1 and/or Hmcn2. Furthermore, given the similarity of phenotypes between zebrafish *fras1*, *hmcn1* mutants and *fbn2* mutants, coupled with the lack of mutations in any of the *FRAS1*/*FREM*/*GRIP* genes in approximately half of Fraser patients, we consider *HMCN1* and *FBN2* to be strong candidate genes mutated in these patients. Other candidates emerging from our work are Furin proprotein convertases.

### Itgα3 and Lamα5 are primarily required for epidermis-BM attachment; Fras1, Frem, and Hmcn1 for BM-dermis attachment and possibly BM elasticity

**Figure 10:**
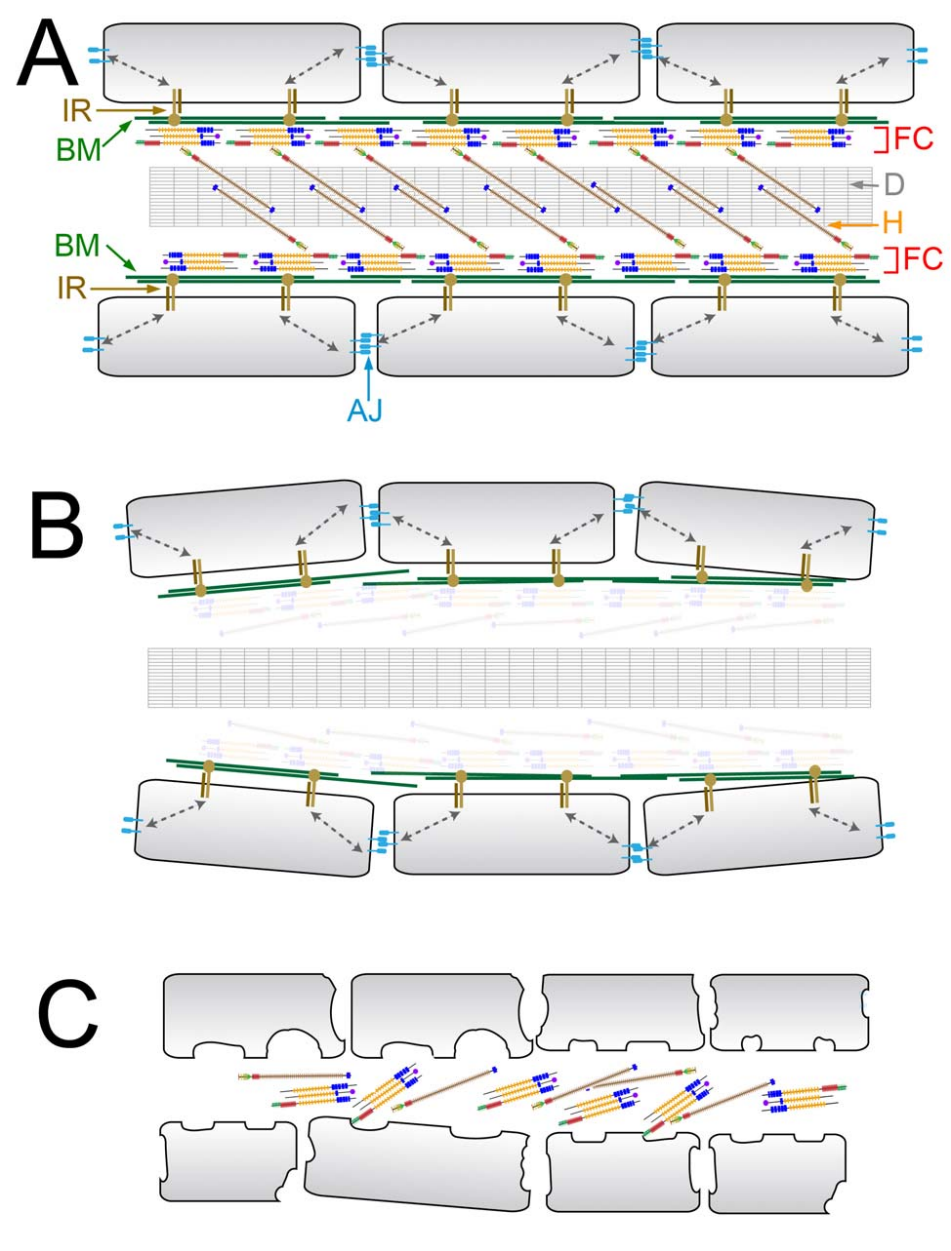
Model describing the ultrastructural changes underlying the fin phenotypes of blistering and dysmorphogenesis mutants. (A) A simplified model depicting epidermis–basement membrane (BM) and dermis attachments in wild-type embryo. A transverse section through a fin shows basal epidermal cells (grey boxes) attached via Integrin heterodimeric receptors (IR, light and dark brown lines) to the underlying BM (dark green lines) containing Lamininα5. Below this lies the Fraser complex (FC, indicated by a red bracket) containing Fras1, Frem2(a/b)/3, and Frem1(a/b). This attaches the BM to the underlying dermis (D, grey grid) possibly via a complex including Hemicentin1 (H, indicated with an orange arrow). Cell–cell adhesion between epidermal cells is maintained through adherens junctions (AJ, light blue lollipops), which assemble and are stabilised via Integrin receptor signalling from the basal side (grey dashed arrows). (B) Loss of any component of the Fraser complex or Hemicentin1 abrogates attachment of the BM to the underlying dermis. However, Integrin-mediated cell attachment of epidermal cells to the BM is not compromised, and Integrin-signalling continues to maintain adherens junctions and high cell–cell adhesion among epidermal cells. As a consequence, the fin fold lifts away from the underlying dermis as an intact epidermal sheet, causing blistering. (C) Loss of the Integrin receptor in epidermal cells or its ligand (Laminin) in the basement membrane leads to compromised epidermal–BM attachment, and to reduced Integrin signalling within epidermal cells, thereby also compromising intracellular Integrin signalling, adherens junctions and epidermal cell-cell adhesion). As a result, the integrity of the epidermal sheet is disrupted, and the sheets fail to undergo normal fin morphogenesis (compare with schematic shown in Ref. 21).

In addition to mutants displaying blistering of the fins, we also described a second class of mutants displaying globally compromised fin morphogenesis. One of them, *fransen*, is caused by a mutation in the Lamininα5, a subunit of Laminin511, which like Fras1/Frem2 proteins and Hmcn1 is integral part of the basement membrane (BM). The other, *badfin*, is caused by a mutation in Integrinα3, which is part of the α3β1 Integrin dimer, the receptor for Laminin511 and other BM proteins on epidermal cells. Similarly, Frem1 has been shown to mediate cellular adhesion in vitro through interactions with α5 and α8-containing integrin receptors [43]. We can only speculate about the molecular basis of the different phenotypes of *fras1*/*frem*/*hmcn1* (fin blistering) versus *lama5/itga5* mutants (compromised fin morphogenesis). Recent studies of another *lama5* allele have revealed defects in epidermal integrity of the fins, including compromised epidermal cell-cell adhesion and compromised attachment of the epidermis in the underlying BM [21]. In contrast, we could show here by electron microscopy that cell-cell adhesion and cell-BM attachment remains intact in the *fras1* and *hmcn1* fin blister mutants. This suggests that Lamα5 and Fras1/Frem/Hmcn1 are required in different layers of the BM, with Lamα5 primarily involved with epidermis-BM attachment via an Integrinα3 containing receptor, whereas Fras1/Frem2/Hmcn1 acting in deeper positions below the BM, mediating BM-dermis attachment. The retention of the BM to the cell surface in the fin blistering mutants has important implications for the cells. As Laminin activation of Integrins still occurs, we could expect Integrin-mediated outside-in signalling to persist. One such known intracellular effect downstream of Integrinα3 signals is the assembly of adherens junctions, which are crucial for proper cell-cell adhesion [44]. Thus the critical difference between the *fras1*/*frem*/*hmcn1* mutants and the *itga3*/*lama5* mutants is the maintenance of Integrin-mediated signalling via basement membrane components on the basal side of epidermal cells in the blistering mutants, promoting persistent strong cell-cell adhesion through adherens junction at the lateral sides of the cells (summarised in Figure 10). It is also noteworthy that in contrast to many Laminins and Collagens, the proteins of the Fras1/Frem2 complex as well as Hmcn1 and Fbn2 are no constitutive BM components. Rather, their occurrence is restricted to particular embryonic sites and developmental stages, such as the apical fin folds during fin morphogenesis. According to our EM analyses, basement membranes at this site and stage are just beginning to become morphologically distinct, suggesting that Fras1/Frem2/Hmcn1/Fbn2 might be specifically required during basement membrane formation. In addition or alternatively, they might confer specific properties such as elasticity to basement membranes that are under high mechanical stress or in the process of spatial rearrangements, as during fin or limb outgrowth. This would also be in line with the formerly described attachment of Fibulins and Fibrillins to elastic fibers [45], [46], [47].

## Materials and Methods

### Fish lines

Embryos were obtained through natural crosses and staged according to [48]. The mutant alleles *pif^te262^, pif^tm95^, nel^tq207^, bla^ta90^, fra^tc17^*, *rfl^tc280b^*, *rfl^tr240^*, *bdf^tz296^* and *stu^td204e^* were obtained from the Tübingen stock centre and have been previously described [13], [26], whilst the alleles *bdf^ fr21^*, *nel^fr22^* and *rfl^fr23^* were isolated in a recent ENU mutagenesis screen conducted in the Hammerschmidt laboratory in Freiburg. *pif^b1048^* and *pif^b1130^* were isolated in another recent ENU mutagenesis screen conducted in the Kimmel laboratory. Meitoic mapping was performed by crossing heterozygous adults to the wild-type WIK strain to generate hybrid F1 mapping fish.

### Genetic mapping

Genetic mapping was performed largely as per [49]. Heterozygous F1 carriers from WIK out-crosses were in-crossed and pools of either mutant or sibling F2 progeny were subjected to bulk segregation single sequence linkage polymorphism (SSLP) analysis. Upon assignment to a linkage group, fine SSLP mapping on single arrayed mutant embryos was used to confirm linkage and generate a broad interval on the genome. Candidate genes within this interval were selected and tested for expression in the fin fold and further analysis.

### Microscopy

For imaging, live embryos were anesthetised with Tricaine and mounted in 3% methyl cellulose, whilst embryos stained by in situ hybridisation or antibody staining were cleared in glycerol prior to mounting. Fluorescent images were taken with a Zeiss Confocal microscope (LSM710 META); bright-field or Nomarski microscopy was performed on a Zeiss Axioimager. Transmission electron microscopy was carried out as previously described [50].

### In situ hybridisations and probe synthesis

Embryos were fixed in 4% PFA in PBS overnight at 4°C and in situ hybridisations were performed as previously described [51], using probes generated from cloned cDNA fragments of *fras1, frem2a, frem2b, frem3, frem1a, frem1b, grip1, grip2, hmcn1, hmcn2, furina, fibrillin2* and *itga3*. Probes were synthesised from linearised plasmids using the Roche digoxygenin RNA synthesis kit.

### Antibody generation, immunohistochemistry, and sectioning

An antibody against zebrafish Fras1 was generated by cloning the cDNA region encoding amino acids A1210 to H1525 of the Fras1 protein (predicted size: 34.5 kDa) into the prokaryotic expression vector pGEX-2TK-P (GE Heathcare) containing a glutathione S-transferase (GST) tag. This fragment corresponds to that used by Vrontou et al. to generate a specific antibody against mouse Fras1 [4]. Recombinant protein was expressed in E. coli BL21 cells, purified via glutathione affinity chromatography, and used to immunise rabbits (Pineda Antikörper Service, Berlin, Germany). Obtained sera were tested for immunogenicity by western blot and ELISA analysis. Immunoreactive sera were affinity-purified against the same recombinant Fras1 fragment used for immunization, coupled to CNBr-activated sepharose.

Whole mount fluorescent antibody stainings were performed as described [51]. Antibodies and dilutions used were as follows: rabbit anti-zebrafish Fras1 (1∶100); 4A4 anti-p63 (1∶200, Santa Cruz), chicken anti-GFP (1∶200, Invitrogen), AlexaFluor546 goat anti-mouse (1∶400, Invitrogen), AlexaFluor488 goat anti-rabbit (1∶400, Invitrogen) and AlexaFluor 647 goat anti-chicken (1∶200).

For sectioning, double- or triple-immunostained (Fras1, p63, GFP) embryos were counterstained with DAPI to visualise the nuclear DNA, mounted in Durcupan ACM (Fluka Chemicals), cut into 7 µm sections, and analyzed via confocal microscopy.

### Genomic DNA extraction, RNA isolation, cDNA synthesis, RT–PCR, and 5′RACE

Genomic DNA from adult fin or embryos was extracted by incubation of the tissue in lysis buffer for at least 4 hours at 55°C. Extracted DNA was diluted ten-fold before PCR analysis. Total RNA was isolated from embryos using Trizol-LS (Invitrogen, CA) and cDNA synthesized with SuperscriptII reverse transcriptase (Invitrogen). Sequences corresponding to zebrafish orthologues of *fras1, frem2a, hmcn1, lama5* and *itga3* were obtained from the zebrafish genome (Ensembl, Sanger Center), and amplified via RT-PCR. To determine the full 5′ sequence of *fras1, frem2a, frem2b, frem3, frem1a, frem1b, grip1, grip2, hmcn1* and *hmcn2* cDNAs, 5′RACE was performed using the SMART RACE kit (BD Biosciences, CA).

### Morpholino (MO) injections

Morpholinos were ordered from Gene Tools (Philomath, OR) and dissolved in distilled water to 1 mM stock solutions. For injection, stocks were diluted in Danieau's buffer and Phenol Red as indicated in text, tables or figures [52]. 1.5 nl of MO solution was injected into embryos at the 1–4 cell stage using glass needles pulled on a Sutter needle puller and a Nanoject injection apparatus (Word Precision Instruments). MOs used and their sequences (given 5′-3′) were as follows:

*fras1-ATG*: ATAGGACCCATATTCACTTAAAAGC

*fras1-splice*: CTTTGGTGTGCTATAAAAAATTGAA

*frem1a-ATG*: CACATTTGCTGGTTTTTACAGTCAT

*frem1a-splice*: TATAATGTGATGCTTGTTACCCAGC

*frem1b-ATG*: GGAAGAAAACCCCCATCTTTTTGGC

*frem1b-splice* AGCAGATGCTGGTCATTTACATGTC

*frem2a-ATG*: GGAGAAGAAATCTGTGAAGTTCCAT

*frem2b-ATG*: GCTCTGTTCTACTCCCAGCCATTTG

*frem2b-5′UTR* CATTTGTAATGTAAACAACAGTTAC

*frem3-ATG*: GCAGACAACCAGCCATATCTACAGC

*frem3-splice* AGATGATGGTCTCTGACCTGTGTCT

*grip1-ATG*: TGACAAAGCCAAGAAAGCGTTCCAT

*grip1-splice* AATGCGTCACTTGTACTGACCTAGC

*grip2-ATG*: CTCTCTCCTCAAACCACACAGCATC

*grip2-5′UTR* ATCGTGGGAAAATCACGAATCCATT

*hmcn1-ATG*: AAAACGGCGAAGTTATCAAGTCCAT

*hmcn2-ATG*: TAACGACAAACTTTTTCATTCTCAC

*hmcn2-splice*: GTTGTGCTGATGTAGTAATACCTTT

*lama5-splice*: AACGCTTAGTTGGCACCTTGTTGGC

*itga3-ATG*: GTGCAGAGACTTTCCGGCCATATTT

*itga3*-splice AGTCAAATGCGCTAACTCACCCTGC

*furina-ATG*: TATAGGAGAACCAAGGCAGGAATT

*fbn2-splice*: AGTTTTATTGTGAACTCACCCACAC

### Cell transplantations

For the analysis of Fras1 protein distribution behaviour in vivo (Figure 7 K and 7L), chimeric embryos were generated by injecting wild-type embryos with in vitro synthesised *GFP* mRNA, or embryos from a clutch of two *stu*/+ parents with *furina* MO (1∶20 dilution of 1 mM stock) and *GFP* mRNA, followed by homochronic and homotopic transplantation of ventral ectodermal cells into the offspring of two *pif^te262^*/+ parents at the shield stage. Chimeric embryos were inspected for the *pif* phenotype and for fluorescent fin epidermal cells at 26 hpf, and were processed via immunostainings and sectioning as described above. In case of donors from a *stu*/+ x *stu*/+ cross, donor embryos were genotyped after the transplantation as previously described [19], [26]. For cell lineage analysis (Figure 7Q and 7R), similar transplantations were carried out between *Tg(bactin::hras-egfp)* (*vu119*; [53]) donors and wild-type hosts.

### Fras1 and Frem2 ectodomain shedding assay

The assay was conducted as previously described [23]. Briefly, 293F cells were transfected with HA-tagged mouse Fras1, Myc-tagged mouse Frem2 or empty expression vector, and incubated for 6 hours. Then either DMSO or the Furin inhibitor Decanoyl-RVKR-CMK (Calbiochem) was added to the cells to a final concentration of 30 µM. After further incubation for 24 hours, levels of protein shed into the medium were determined by western immunoblotting of both cell lysates (as a reference) and conditioned medium, using antibodies against the corresponding tags. 293F cells are derivatives of HEK-293 cells, which are known to express Furin endogenously [54], [55].

## Supporting Information

Figure S1 Zebrafish fin mutants can be classed in two phenotypic groups. (A–X) Lateral Normarski images of the mutants *pinfin* (B–C, J–K, R–S), *blasen^ta90^* (D, L, T), *rafels^fr23^* (E,M,U), *nagel^tq207^* (F, N, V), *fransen^tc17^* (G, O, W) and *badfin^fr21^* (H, P, X) displaying medial fin defects compared to wild-type embryos (A,I,Q). Embryos are shown at 32 hpf (A–H), 48 hpf (I–P) and 5 dpf (Q–X). Images of both a strong (*pif^te262^*; B, J, R) and a weak (*pif^tm95^*; C, K, S) allele of *pinfin* are shown, which along with *blasen*, *rafels* and *nagel,* all display blisters within the fin fold. In contrast *fransen* and *badfin* mutants do not show blisters, rather dysmorphogenesis from 32 hpf onwards. Within the group of blister mutants, there was a range of severity, such that the blisters of the weak *pinfin* allele (*pif^tm95^*) and the single *blasen* allele (*bla^ta90^*) appear quite small at 30 hpf and are only visible in some mutant embryos at 48 hpf, whilst the blisters of the strongest *pinfin* allele (*pif^te262^*) are prominent at 30 hpf and always visible at 48 hpf. The blisters of the *rafels* alleles are clear but only prominent in the posterior medial fin at 48 hpf and not evident at 32 hpf. The extent of fin fold degeneration is more severe at 5 dpf in the strong *pinfin* allele than either the *blasen* or weak *pinfin* allele. All three *nagel* alleles display large blisters covering much of the fin fold field, but rarely affect the blood islands. Like the strong *pinfin* allele, these blisters persist until 48 hpf at which point the fin begins to degenerate. (Y–Z) Uniquely *pif^tm95/+^* heterozygous embryos display a mild fin blister phenotype (Z-arrow) seen in either a lateral view (top panel) or dorsal view (lower panel) and compared to a WT sibling (Y).
(4.37 MB TIF)

Figure S2 Pectoral and adult tail fin phenotypes. (A–I) Lateral views of pectoral fins at 72 hpf showing the blisters (red arrows) present in *pif^te262/te262^* (B), *pif^tm95/ttm95^* (C), *bla^ta90/ta90^* (D), *nel^tq207/tq207^* (E), *stu^td204/td204^* (F) and *rfl^fr23/fr23^* (G) embryos compared to WT (A). The dysmorphogenesis of the pectoral pins in *fra^tc17/tc17^* (H) and *bdf^fr21/fr21^* (I) is highlighted with the edge of the fin circumscribed by red dashed line, clearly showing the reduction of the fin compared to WT (A). (J–O) Adult fin phenotypes at 96 dpf: the tail fins of *pif^tm95/ttm95^* (K) and *bdf^fr21/fr21^* (O) mutants are reduced compared to WT (J), *bla^ta90/ta90^* (L), *nel^tq207/tq207^* (M) and *rfl^fr23/fr23^* (N).
(3.46 MB TIF)

Figure S3 Synergistic interaction and compound mutant/morphant analyses. (A–T) Demonstration of synergistic interactions between *itga3* and *lama5* (A–D), *fras1* and *frem2a* (E–H), *hmcn1* and *fbn2* (I–L), and *fras1* and *hmcn1* (M–P), but not *hmcn1* and *lama5* (Q–T). Images show lateral views of embryos tails at 30 hpf (I–L), 36 hpf (E–H) or 48 hpf (A–D; M–T) after injection of sub-phenotypic doses of morpholinos against *itga3* (B), *fras1* (F,N), *frem2a* (G), *hmcn1* (J,O,S), *fbn2* (K) or *lama5* (C,R). All single morphants appear as their uninjected WT controls (A,E,I,M,Q). In contrast, a dysmorphogenic phenotype is seen upon *itga3* and *lama5* co-injection (D), whilst blisters are evident upon combined injection of *fras1* and *frem2a* (H), *hmcn1* and *fbn2* (L) and *fras1* and *hmcn1* (P). Neither phenotype is seen upon co-injection of *hmcn1* and *lama5* (T). (U-X) Lateral views of the medial fins of *pif^te262/te262^* (V), *nel^tq207/tq207^* (W), *pif^+/te262^; nel^+/tq207^* (U) and *pif^te262/te262^; nel^tq207/tq207^* (X) embryos at 30 hpf, showing that blisters in the double mutant are more severe than in *nel^tq207/tq207^*, but of equal severity to *pif^te262/te262^*. (Y-AB) Lateral views on tails of embryos at 32 hpf, demonstrating that the defects due to triple loss of *frem2a/2b/3* combined with loss of *fras1* (AB) appears as severe as loss of either *fras1* alone (Z) or combined loss of *frem2a/2b/3* (AA). WT embryo is shown for comparison (Y). (AC-AF) Additive function of *hmcn1* and *fbn2* as assessed by generation of compound mutant/strong morphant embryos imaged at 32 hpf. Injection of strong doses of *fbn2* MO into *nel^tq207/tq207^* (AF) generated embryos with stronger blistering than *nel^tq207/tq207^* (AD) or strong *fbn2* morphants (AE) alone.
(4.53 MB TIF)

Figure S4 Confirmation of morphant phenotypes with second morpholino. Injection of second non-overlapping morpholinos was used to verify morphant phenotypes. Injection of translation-blocking morpholinos against *fras1* (A), *frem1a* (B) and *frem2b* (D) into WT embryos realised blisters in the fin fold comparable to those seen with the original morpholinos. The fin blister phenotypes of *rafels* (C) and *blasen* (E) could be enhanced by the injection of morpholinos targeting the *frem1b* 5′UTR and *frem3* ATG respectively. Co-injection of *grip1* ATG and *grip2* 5′UTR morpholinos also yield strong blistering of the fin fold identical to that obtained with the original MOs (F).
(1.88 MB TIF)

Figure S5 Fras1 distribution is compromised in *sturgeon* mutant fins. Transverse sections of WT (A) or *stu^−/−^* (B) posterior medial fins at 32 hpf, fluorescently immunostained for Fras1 (green), p63 (pink) and DAPI (blue). The extent of relative proximal extension of Fras1 protein appears reduced in *stu^−/−^* embryos (B) compared to WT (A). Extent of Fras1 staining is delineated by adjacent white line. Note that in the mutant, levels of Fras1 protein in the smaller domain appear correspondingly higher.
(0.74 MB TIF)

Figure S6 Nature of molecular lesions in *nel* alleles. (A–B) Sequence chromatograms of *hmcn1* cDNA from *nel^tq207/tq207^* (A) and *nel^fr22/fr22^* (B) are shown with mutant sequence given below the WT sequence above. Whilst the *nel^tq207^* allele displays a nonsense mutation, the *nel^fr22^* allele showed double peaks from nucleotide 8538 onwards, suggestive of aberrant splicing. One of the two transcripts generated appears as if spliced at sites used in the WT allele and the other lacked the last 20 bp of exon 55 suggesting generation of a novel splice site 20 bp further upstream from the normal end of exon 55. (C) Sequencing the genomic region around the end of exon 55 in *nel^fr22/fr22^* mutants revealed a T>A transversion within the coding region and 17 bp upstream of the end of exon 55 (8541T>A). This has a two-fold effect, generating a nonsense mutation Y2847* and secondly converting the genomic sequence from 8538-gtat-8541 sequence to 8538-gtaa-8541, a consensus splice site. (D–G) Diagrams of the aberrant splicing at the end of exon 55 in WT and *nel^fr22/fr22^* mutants. In WT embryos, splicing occurs at the nucleotides shown (D; exon sequence is given in uppercase, intron sequence in lowecase.) to yield the sole WT transcript (E; partial sequence given with the translation below). In the mutant, two transcripts (denoted M1 and M2) are generated, M1 occurring at the WT location, and a second, M2, generated due to a novel splice donor consensus sequence (F). The cDNA sequences of both M1 and M2 are given with the translation below (G). In M1 the T>A yields an in frame nonsense mutation whilst the same mutation also leads to some splicing giving rise to the M2 transcript which deletes the last 20 nt of exon 55, creating a frameshift which would be predicted to introduce 10 erroneous amino acids followed by a stop codon.
(0.60 MB TIF)
