# Supplementary figures and images for: Genetic Analysis of Fin Development in Zebrafish Identifies Furin and Hemicentin1 as Potential Novel Fraser Syndrome Disease Genes
Source: PLoS Genet. 2010 Apr 15;6(4):e1000907. doi: 10.1371/journal.pgen.1000907 (PMC2855323; doi:10.1371/journal.pgen.1000907)

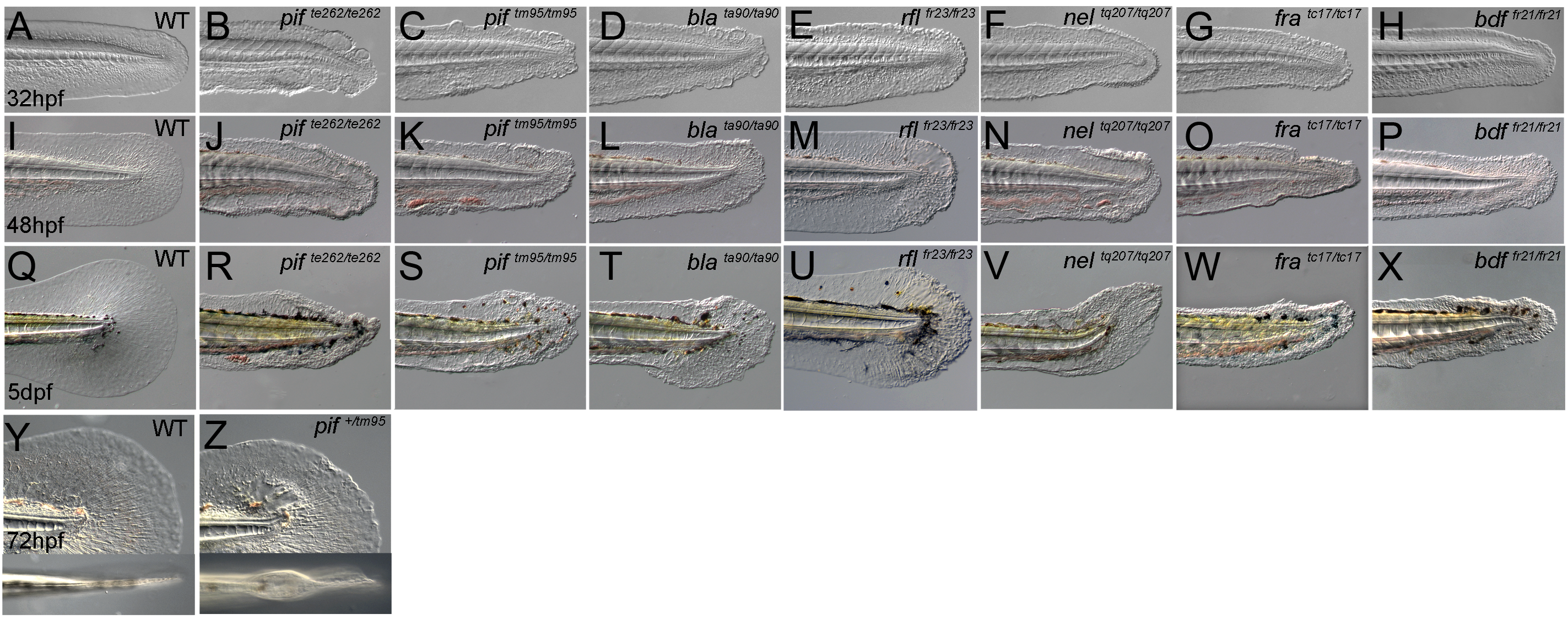

Supplement: Figure S1 — Zebrafish fin mutants can be classed in two phenotypic groups. (A–X) Lateral Normarski images of the mutants pinfin (B–C, J–K, R–S), blasenta90 (D, L, T), rafelsfr23 (E,M,U), nageltq207 (F, N, V), fransentc17 (G, O, W) and badfinfr21 (H, P, X) displaying medial fin defects compared to wild-type embryos (A,I,Q). Embryos are shown at 32 hpf (A–H), 48 hpf (I–P) and 5 dpf (Q–X). Images of both a strong (pifte262; B, J, R) and a weak (piftm95; C, K, S) allele of pinfin are shown, which along with blasen, rafels and nagel, all display blisters within the fin fold. In contrast fransen and badfin mutants do not show blisters, rather dysmorphogenesis from 32 hpf onwards. Within the group of blister mutants, there was a range of severity, such that the blisters of the weak pinfin allele (piftm95) and the single blasen allele (blata90) appear quite small at 30 hpf and are only visible in some mutant embryos at 48 hpf, whilst the blisters of the strongest pinfin allele (pifte262) are prominent at 30 hpf and always visible at 48 hpf. The blisters of the rafels alleles are clear but only prominent in the posterior medial fin at 48 hpf and not evident at 32 hpf. The extent of fin fold degeneration is more severe at 5 dpf in the strong pinfin allele than either the blasen or weak pinfin allele. All three nagel alleles display large blisters covering much of the fin fold field, but rarely affect the blood islands. Like the strong pinfin allele, these blisters persist until 48 hpf at which point the fin begins to degenerate. (Y–Z) Uniquely piftm95/+ heterozygous embryos display a mild fin blister phenotype (Z-arrow) seen in either a lateral view (top panel) or dorsal view (lower panel) and compared to a WT sibling (Y). (4.37 MB TIF) [file pgen.1000907.s001.tif]

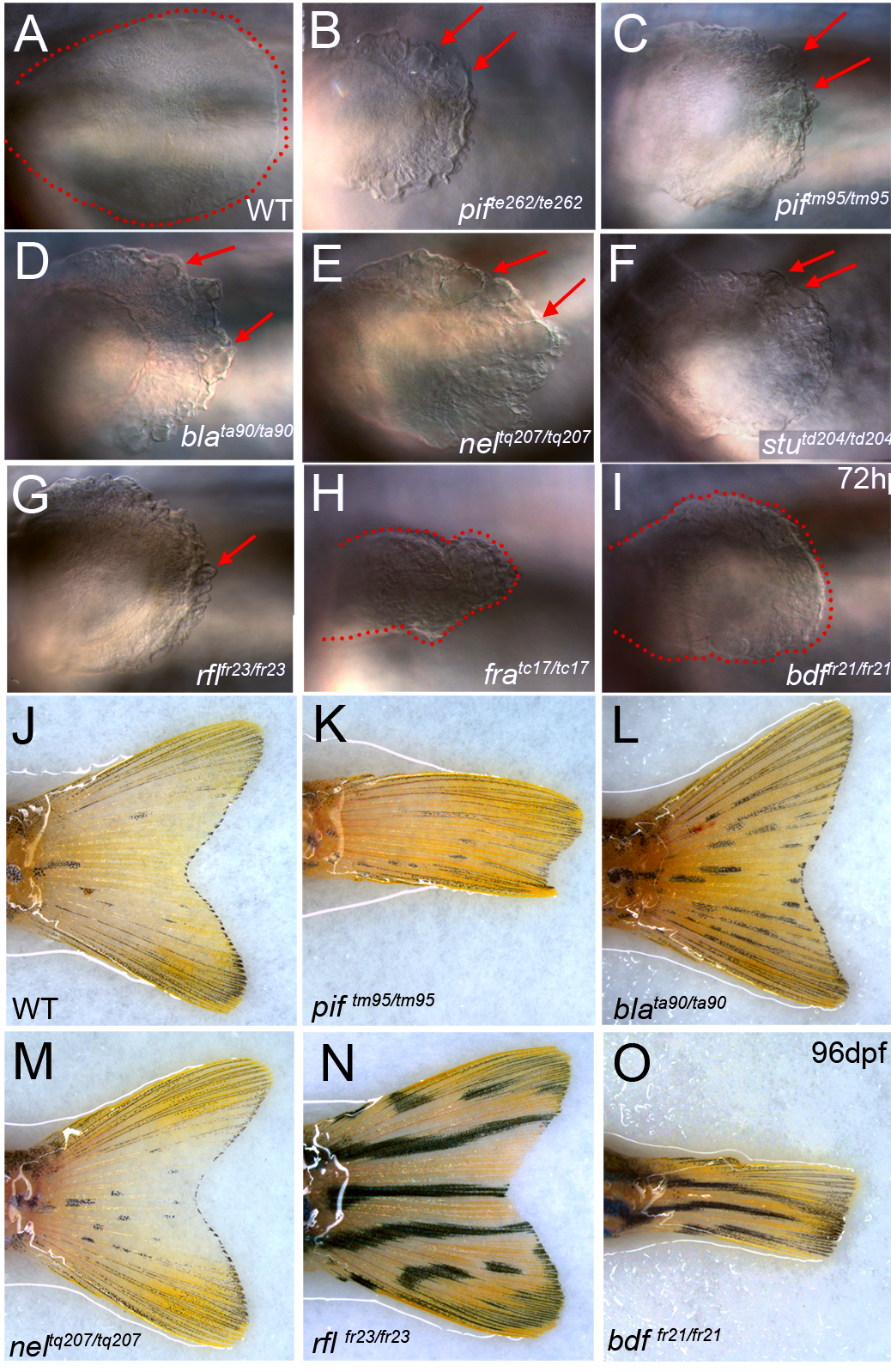

Supplement: Figure S2 — Pectoral and adult tail fin phenotypes. (A–I) Lateral views of pectoral fins at 72 hpf showing the blisters (red arrows) present in pifte262/te262 (B), piftm95/ttm95 (C), blata90/ta90 (D), neltq207/tq207 (E), stutd204/td204 (F) and rflfr23/fr23 (G) embryos compared to WT (A). The dysmorphogenesis of the pectoral pins in fratc17/tc17 (H) and bdffr21/fr21 (I) is highlighted with the edge of the fin circumscribed by red dashed line, clearly showing the reduction of the fin compared to WT (A). (J–O) Adult fin phenotypes at 96 dpf: the tail fins of piftm95/ttm95 (K) and bdffr21/fr21 (O) mutants are reduced compared to WT (J), blata90/ta90 (L), neltq207/tq207 (M) and rflfr23/fr23 (N). (3.46 MB TIF) [file pgen.1000907.s002.tif]

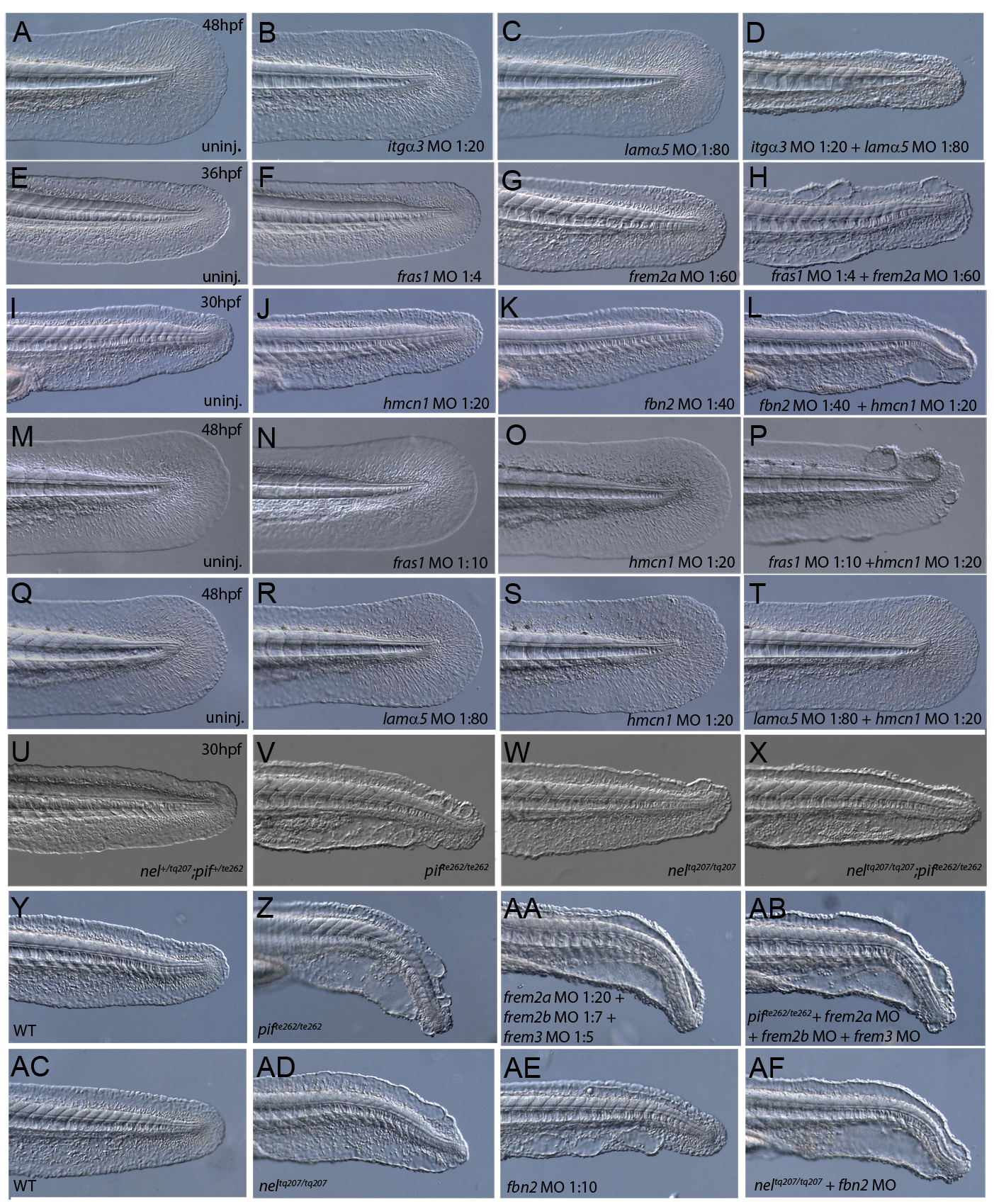

Supplement: Figure S3 — Synergistic interaction and compound mutant/morphant analyses. (A–T) Demonstration of synergistic interactions between itga3 and lama5 (A–D), fras1 and frem2a (E–H), hmcn1 and fbn2 (I–L), and fras1 and hmcn1 (M–P), but not hmcn1 and lama5 (Q–T). Images show lateral views of embryos tails at 30 hpf (I–L), 36 hpf (E–H) or 48 hpf (A–D; M–T) after injection of sub-phenotypic doses of morpholinos against itga3 (B), fras1 (F,N), frem2a (G), hmcn1 (J,O,S), fbn2 (K) or lama5 (C,R). All single morphants appear as their uninjected WT controls (A,E,I,M,Q). In contrast, a dysmorphogenic phenotype is seen upon itga3 and lama5 co-injection (D), whilst blisters are evident upon combined injection of fras1 and frem2a (H), hmcn1 and fbn2 (L) and fras1 and hmcn1 (P). Neither phenotype is seen upon co-injection of hmcn1 and lama5 (T). (U-X) Lateral views of the medial fins of pifte262/te262 (V), neltq207/tq207 (W), pif+/te262; nel+/tq207 (U) and pifte262/te262; neltq207/tq207 (X) embryos at 30 hpf, showing that blisters in the double mutant are more severe than in neltq207/tq207, but of equal severity to pifte262/te262. (Y-AB) Lateral views on tails of embryos at 32 hpf, demonstrating that the defects due to triple loss of frem2a/2b/3 combined with loss of fras1 (AB) appears as severe as loss of either fras1 alone (Z) or combined loss of frem2a/2b/3 (AA). WT embryo is shown for comparison (Y). (AC-AF) Additive function of hmcn1 and fbn2 as assessed by generation of compound mutant/strong morphant embryos imaged at 32 hpf. Injection of strong doses of fbn2 MO into neltq207/tq207 (AF) generated embryos with stronger blistering than neltq207/tq207 (AD) or strong fbn2 morphants (AE) alone. (4.53 MB TIF) [file pgen.1000907.s003.tif]

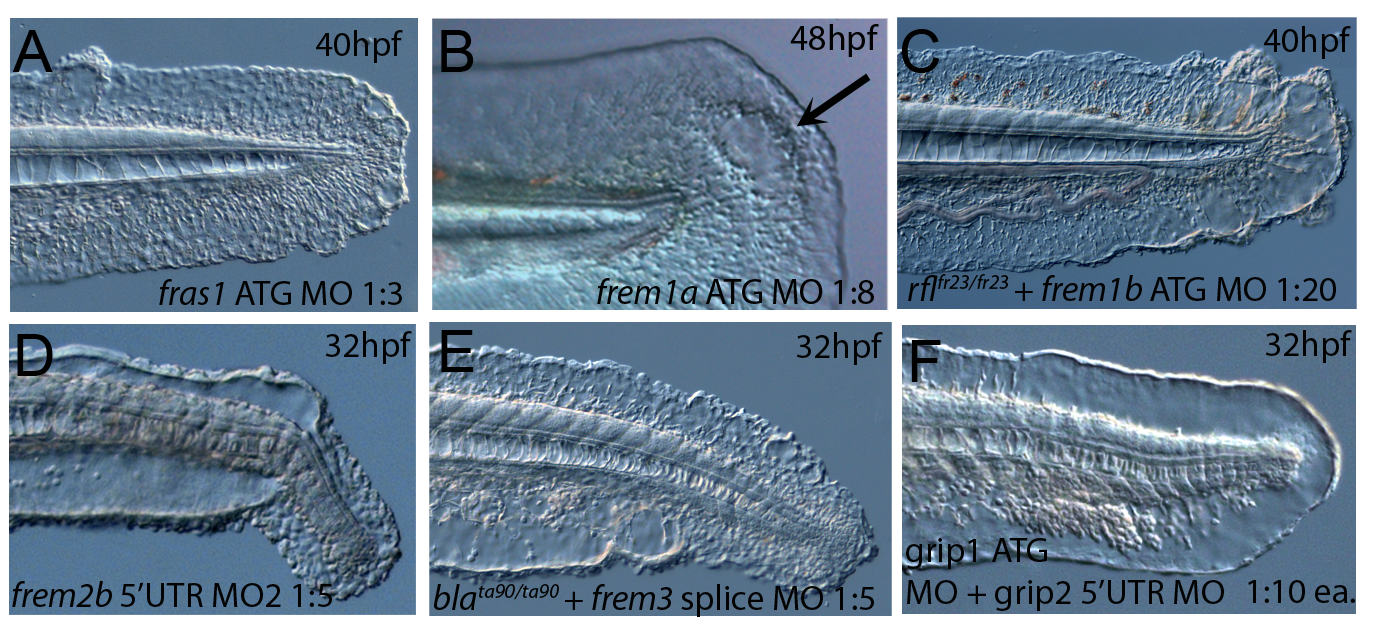

Supplement: Figure S4 — Confirmation of morphant phenotypes with second morpholino. Injection of second non-overlapping morpholinos was used to verify morphant phenotypes. Injection of translation-blocking morpholinos against fras1 (A), frem1a (B) and frem2b (D) into WT embryos realised blisters in the fin fold comparable to those seen with the original morpholinos. The fin blister phenotypes of rafels (C) and blasen (E) could be enhanced by the injection of morpholinos targeting the frem1b 5′UTR and frem3 ATG respectively. Co-injection of grip1 ATG and grip2 5′UTR morpholinos also yield strong blistering of the fin fold identical to that obtained with the original MOs (F). (1.88 MB TIF) [file pgen.1000907.s004.tif]

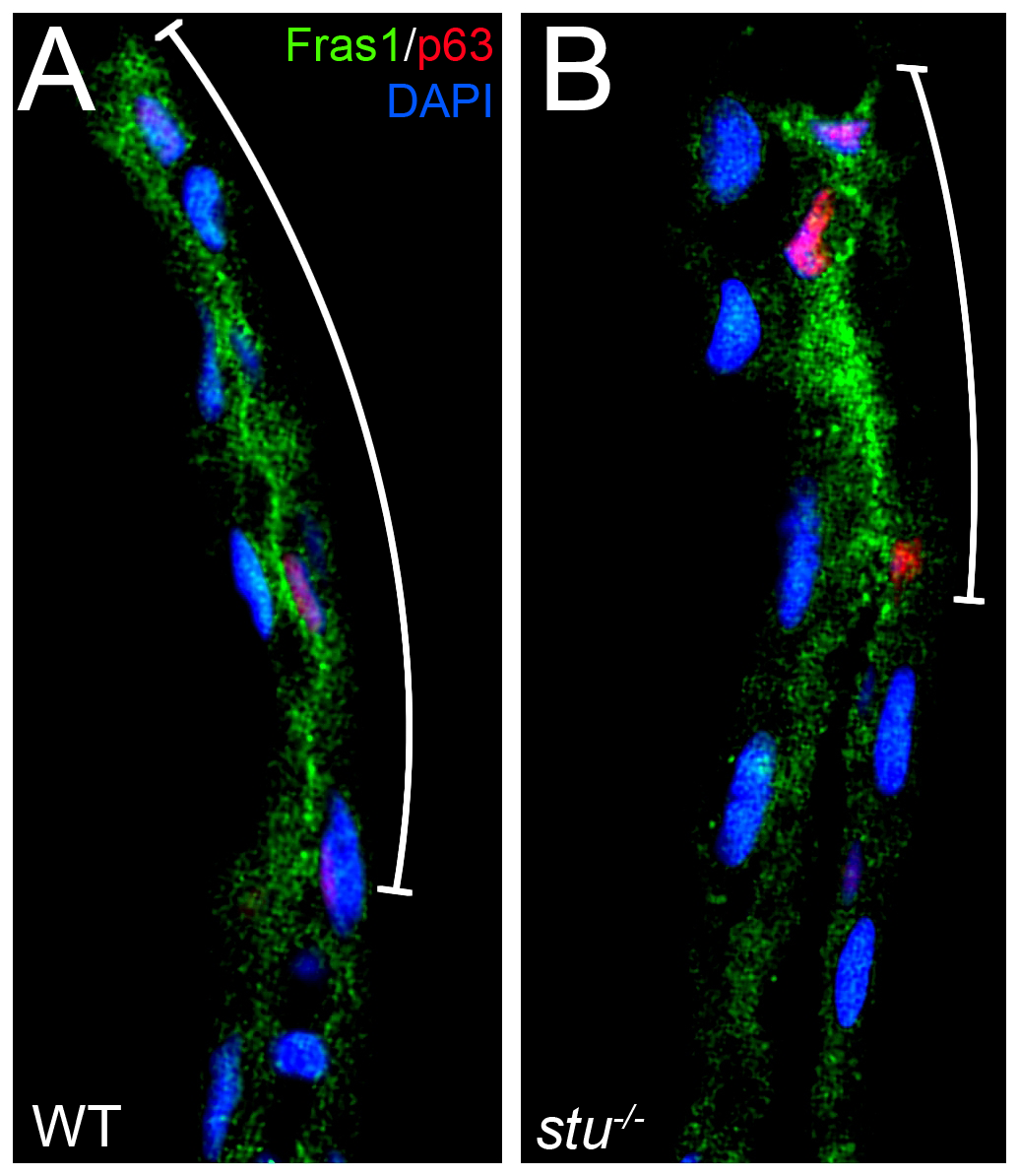

Supplement: Figure S5 — Fras1 distribution is compromised in sturgeon mutant fins. Transverse sections of WT (A) or stu−/− (B) posterior medial fins at 32 hpf, fluorescently immunostained for Fras1 (green), p63 (pink) and DAPI (blue). The extent of relative proximal extension of Fras1 protein appears reduced in stu−/− embryos (B) compared to WT (A). Extent of Fras1 staining is delineated by adjacent white line. Note that in the mutant, levels of Fras1 protein in the smaller domain appear correspondingly higher. (0.74 MB TIF) [file pgen.1000907.s005.tif]

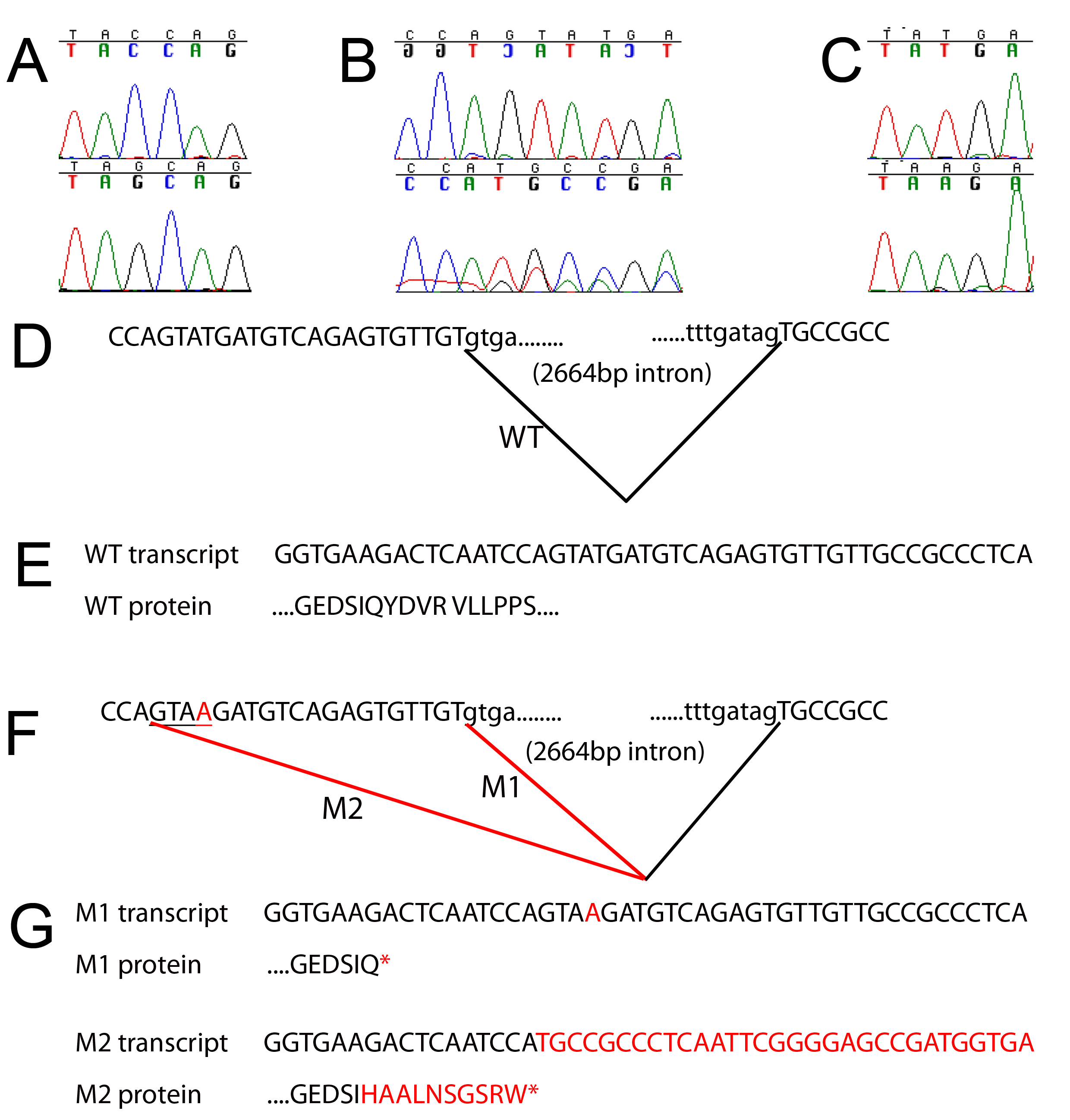

Supplement: Figure S6 — Nature of molecular lesions in nel alleles. (A–B) Sequence chromatograms of hmcn1 cDNA from neltq207/tq207 (A) and nelfr22/fr22 (B) are shown with mutant sequence given below the WT sequence above. Whilst the neltq207 allele displays a nonsense mutation, the nelfr22 allele showed double peaks from nucleotide 8538 onwards, suggestive of aberrant splicing. One of the two transcripts generated appears as if spliced at sites used in the WT allele and the other lacked the last 20 bp of exon 55 suggesting generation of a novel splice site 20 bp further upstream from the normal end of exon 55. (C) Sequencing the genomic region around the end of exon 55 in nelfr22/fr22 mutants revealed a T>A transversion within the coding region and 17 bp upstream of the end of exon 55 (8541T>A). This has a two-fold effect, generating a nonsense mutation Y2847* and secondly converting the genomic sequence from 8538-gtat-8541 sequence to 8538-gtaa-8541, a consensus splice site. (D–G) Diagrams of the aberrant splicing at the end of exon 55 in WT and nelfr22/fr22 mutants. In WT embryos, splicing occurs at the nucleotides shown (D; exon sequence is given in uppercase, intron sequence in lowecase.) to yield the sole WT transcript (E; partial sequence given with the translation below). In the mutant, two transcripts (denoted M1 and M2) are generated, M1 occurring at the WT location, and a second, M2, generated due to a novel splice donor consensus sequence (F). The cDNA sequences of both M1 and M2 are given with the translation below (G). In M1 the T>A yields an in frame nonsense mutation whilst the same mutation also leads to some splicing giving rise to the M2 transcript which deletes the last 20 nt of exon 55, creating a frameshift which would be predicted to introduce 10 erroneous amino acids followed by a stop codon. (0.60 MB TIF) [file pgen.1000907.s006.tif]
